# Biological treatments for co-occurring eating disorders and psychological trauma: a systematic review

**DOI:** 10.3389/fpsyt.2025.1523269

**Published:** 2025-02-21

**Authors:** Ella van Beers, Irene de Vries, Caroline Planting, Carolien Christ, Edwin de Beurs, Elske van den Berg

**Affiliations:** ^1^ Novarum Center for Eating Disorders & Obesity, Amstelveen, Netherlands; ^2^ Department of Clinical Psychology, Leiden University, Leiden, Netherlands; ^3^ Department of Psychiatry, Amsterdam University Medical Center, Vrije Universiteit, Amsterdam, Netherlands; ^4^ Department Medical Library, GGZ inGeest Specialized Mental Health Care, Amsterdam, Netherlands; ^5^ Research Department, Arkin Mental Health Institute, Amsterdam, Netherlands

**Keywords:** systematic review, eating disorder, post-traumatic stress disorder, ketamine, rTMS, MDMA, deep brain stimulation

## Abstract

**Introduction:**

Many people with eating disorders report having experienced childhood maltreatment or a traumatic event prior to developing an eating disorder. Although many people with eating disorders have significant traumatic exposure or symptoms of post-traumatic stress disorder, very little research has examined the effects of combined treatments for this group. The purpose of this systematic review was to synthesize all existing research on biological treatments for those with eating disorders and psychological trauma, evaluate their safety, and identify future areas of research in this area to support patients with eating disorders and psychological trauma.

**Method:**

A multi-step literature search, according to an *a priori* protocol was performed on PubMed, Embase, APA PsycINFO, Web of Science, Scopus and Cochrane Central. Studies needed to include a biological intervention and report on at least one eating disorder or psychological trauma outcome. Given the limited research in this area, minimal exclusion criteria were applied. A quality assessment of all included studies was completed using the Risk of Bias in Non-Randomized Studies-or Interventions (ROBINS-I) tool.

**Results:**

After removing duplicates, 2623 article titles and abstracts were screened, with 43 articles selected for a full-text review. Following the full-text review, 11 articles met the inclusion criteria. The biological treatments examined included repurposed medications (n = 3), ketamine (n = 2), repetitive transcranial magnetic stimulation (rTMS; n = 2), deep brain stimulation (n =1) electroconvulsive therapy (ECT; n = 1), 3,4-methylenedioxymethamphetamine (MDMA; n = 1), and neurofeedback (n = 1). All studies reported on some improvement in either eating disorder or trauma pathology, with the strongest effect for repetitive transcranial magnetic stimulation and MDMA. While some effects were promising, missing data and selective reporting limited the interpretability of the findings. Adverse events across interventions were common.

**Conclusion:**

Although psychological trauma is common in those with eating disorders, very few treatments have been evaluated in this population. Future work should aim to investigate biological treatments for those with co-occurring eating disorders and psychological trauma, as these evolving treatments show potential benefits for this complex group.

## Introduction

Eating disorders, such as anorexia nervosa, bulimia nervosa, and binge eating disorder, are complex mental illnesses that can develop in response to a combination of biological, psychological, and social stressors. Some factors that can put patients at a greater risk of developing an eating disorder are high levels of perfectionism, a genetic predisposition or family history of eating disorders, bullying, and traumatic life events (1–3). Markedly, many patients with eating disorders report having experienced adverse childhood events before the onset of their eating disorder (4–6). Prior work in this area has found that a history of emotional abuse or neglect, physical abuse, and sexual abuse all significantly increase the risk for developing an eating disorder (OR = 3.3, OR = 3.5, OR = 3.4, and OR = 11.0, respectively; 5), and many patients with eating disorders describe experiencing psychological trauma or have co-occurring post-traumatic stress disorder (PTSD).

While PTSD is common among those with eating disorders, there is a large range in the estimated prevalence of PTSD in eating disorder populations. One study examining 603 patients across the United States found that 43% of those with severe eating disorders met the Diagnostic and Statistical Manual – 5th Edition criteria for PTSD (7, 8). However, a quantitative synthesis of 33 patient samples found the co-occurring prevalence of eating disorders and PTSD was 24.6% when weighted by study quality, and 18.6% when weighted by sample size (9). Despite the discrepancy in these findings, the estimated prevalence of PTSD in patients with eating disorders is more than double that of the general population, which has an estimated PTSD lifetime prevalence of 8.3% (10).

Although there are contrasting findings in this area, many studies have found that co-occurring PTSD puts patients with eating disorder at a higher risk of early treatment termination, relapse, and suicidality (11–14). The recommended treatment for those with eating disorders is cognitive behavioral therapy-enhanced (CBT-E), a highly structured, evidence-based therapy aimed at systematically addressing the maintaining mechanisms of an eating disorder (15, 16). While CBT-E is often effective, relapse is common across all eating disorder diagnoses (17), emphasizing the need for more research into understanding the development and maintenance of both disorders. With a better understanding of both disorders and how they intersect, new treatments and add-on interventions can be implemented to improve treatment outcomes.

Eating disorders and PTSD are highly linked psychologically and biologically. Factors including having another psychiatric comorbidity, a family history of mental illness, and compounding exposure to traumatic life events are all predictors for developing a lifetime eating disorder and PTSD (18–20). Exposure to a psychological trauma, such as childhood maltreatment, has been widely associated with the development of both eating disorders and PTSD (21–23). Childhood maltreatment has also been associated with the development of poor body image, emotion dysregulation, and heightened levels of self-criticism (24, 25), all of which are common in eating disorder populations. Further, binge-eating and self-induced vomiting can develop as a way to manage difficult emotions (26) and cope with intrusive PTSD symptoms such as nightmares or flashbacks (27).

While psychological factors play a key role in the development of eating disorders and PTSD, research is rapidly evolving to investigate the underlying physiology of both disorders, such as how changes in epigenetic, neural circuits, and immune system functioning might contribute to the development and maintenance of eating disorders and PTSD. Advances in techniques including genome sequencing, neuroimaging, and neuroendocrine analysis have promoted further exploration into physiological factors underlying eating disorders and PTSD (28–33), providing a more complete theoretical foundation for developing biological interventions and treatments (34).

Genome-wide association studies have found significant genetic underpinnings that influence metabolism, neurotransmitter activity, synaptic structure, and immune system functioning in eating disorders and PTSD (35, 36). A recent genome-wide association study by Nievergelt et al. (35) which analyzed data from over one million people identified 95 independent loci associated with PTSD. Many of the loci identified are linked to changes in brain structures in the dorsolateral prefrontal cortex, amygdala, and cerebellum, and implicate synaptic plasticity relating to stress circuits and the processing of threats. Further, this study found that many of the loci identified in those with PTSD overlap significantly with other psychiatric disorders including substance use disorders and depressive disorders (35, 37, 38). Additionally, genes affecting stress hormones may be of particular interest given the expression of genes encoding a receptor for corticotropin-releasing hormone, predicted the presence of PTSD (39). There is less genetic research on eating disorders, however a genome-wide association by Watson et al. (40) identified eight novel loci implicating brain expressed genes and metabolism in those with anorexia. While specific genes linked to brain regions in those with eating disorders have not yet been identified, alterations in genetic pathways have similarities to those identified in PTSD (41, 42). Identifying the key brain regions involved in eating disorders and PTSD has provided insight into the mechanism of action for effective interventions targeting these areas, including neurofeedback (43), brain stimulation (44–46), and pharmacological treatments (47).

In addition to genetic associations, biochemical assays and imaging studies have shown overlapping alterations in neurotransmission in eating disorders and PTSD. In particular, the dysregulation of glutamate has been associated with both eating disorders and PTSD (48, 49). These changes may cause excitotoxicity and atrophy of the hippocampus, a key region for memories and fear (48, 50, 51). This can also contribute to hypothalamic-pituitary-adrenal axis (HPA) dysregulation, however the findings between eating disorders and PTSD are inconsistent (48, 52). In a study by Lelli et al. (52) examining a group of patients with eating disorder with and without a history of abuse, those with a history of abuse had baseline cortisol levels lower than the control group, whereas those with anorexia nervosa who had not experienced abuse had baseline cortisol levels significantly higher than the control group. These findings align with prior work in this area, which suggest that malnutrition from anorexia can lead to greater cortisol secretion (53) whereas traumatic exposure can lead to HPA hyporeactivity (54, 55).

Closely linked to HPA and cortisol dysregulation, altered immune system functioning may also underly both eating disorders (56, 57) and PTSD (58). Recently, work on cytokines has suggested those with PTSD have heightened cytokine levels (59, 60), and that chronically low inflammation may be a risk factor for PTSD development (61, 62). Interestingly, one study found that despite participants having heightened baseline cytokine levels, during PTSD treatment their cytokine levels increased further, despite psychological distress decreasing (59). However, these findings are inconsistent with prior work (63), highlighting the complex relationship between immune system functioning and PTSD. In patients with anorexia nervosa, the frequency of genes associated with cytokine signaling is significantly higher compared to controls (56, 57). Those with anorexia often have elevated cytokine signaling, which may be due to malnutrition; when patients with anorexia nervosa restore their weight during eating disorder recovery, cytokine levels often normalize, suggesting a decrease in inflammation (64).

Functional magnetic resonance imaging (fMRI) and intrinsic functional connectivity analyses have also demonstrated that those with eating disorders and PTSD also have changes in brain connectivity and neural circuits. A recent review on resting-state fMRI in eating disorder populations found that those with eating disorders display alterations in brain networks compared with controls, particularly with the cingulate cortex (65), which may underly emotion dysregulation. Alterations in these networks may cause disruptions in self-control, such as obsessive or restrictive eating, or repetitive behaviors, such as binge-eating and self-induced vomiting (66, 67). Similarly, a recent study using fMRI in patients with PTSD showed increased insular connectivity (68, 69), which can also cause problems with impulse control, disrupt mood regulation, and interfere with emotional processing (69).

The shared physiological markers in both eating disorders and PTSD underscore the importance of researching the intersection of both disorders, particularly given the high rate of PTSD and reported adverse childhood events in eating disorder populations. With a deeper understanding of the physiological factors underlying both disorders, interventions can be developed to target a patient’s immune response, neurotransmitter levels, or implicated neural circuits.

Treatments for both eating disorders and psychological trauma are primarily focused on using psychological methods to ameliorate either eating disorder or trauma symptoms, respectively. Because many people with eating disorders have co-occurring psychological trauma, the lack of comprehensive care puts a significant burden on patients, practitioners, and the healthcare system. A recent systematic review by Van Den Berg et al. (70) explored all psychological treatments for underweight patients with co-occurring anorexia nervosa and PTSD, and found only three studies with 13 patients have explored comprehensive psychological treatments for this group. This systematic review underscores the severe lack of treatments for patients struggling with anorexia nervosa and PTSD and highlights the importance of expanding this search to account for all available biological treatments as well. In contrast to eating disorders, the research into effective treatments for PTSD is vast, and many biological treatments have demonstrated significant promise for the effective treatment of PTSD symptoms (71). It is therefore worth investigating whether these biological treatments might also be effective for patients with co-occurring eating disorders and PTSD. Exploring biological treatments is of particular importance given many patients fail to benefit from multiple rounds of intensive and costly psychological treatments, which can lead to the development of a chronic or enduring eating disorder (72).

### Rationale & aim

Because many patients with co-occurring eating disorders and PTSD struggle to complete treatment and experience relapse, it is important to explore biological treatments which may enhance existing psychological treatments (72–74). Research on biological treatments is evolving rapidly; therefore, it is important to provide an up-to-date evaluation of all existing interventions that have been examined in patients with eating disorder and trauma symptoms to date. If these interventions show promise in reducing eating disorder or trauma-related symptoms, future work should aim to evaluate these treatments and establish them as an indicated and evidence-based treatment.

This review has several aims: Firstly, it aims to synthesize all available research on biological treatments for those with both eating disorder and trauma symptoms. Secondly, it aims to evaluate the efficacy and safety of these interventions, and to provide recommendations for their use in clinical practice. Lastly, this review aims to identify gaps in the literature that warrant future research and promote the implementation of biological treatments into standard practice.

## Method

### Search strategy and sources

Using the Preferred Reporting Items for Systematic Review and Meta-Analysis 2020 guidelines (PRISMA; 75), a systematic search was conducted of electronic databases PubMed, Embase, APA PsycINFO, Web of Science, Scopus, and Cochrane Central. The search was completed on May 31st, 2024.

Search concepts and terms were developed by using terms found through a preliminary literature review. Using these keywords, Medical Subject Headings (MeSH) and Embase Subject Headings (EMTREE) terms were used to create search concepts. The concepts “Eating and Feeding Disorders” AND “PTSD - Trauma” OR “Childhood Maltreatment” AND a comprehensive list of “Non-psychological treatments” were combined. Complete search strings are available in Annex A. Search strings were developed and reviewed by authors EvB, EvdB, and CP, accounting for various spellings and terms and changes in descriptions and labels for psychiatric diagnoses and interventions over time. Search strings were then adjusted to account for differences in indexing between electronic databases.

Following the electronic database search, duplicates were removed, and all reports yielded were downloaded and input into Rayyan, a specialized software for systematic review organization and management. Authors EvB and EvdB then independently systematically screened all articles based on title and abstract using the inclusion and exclusion criteria defined below. Once the title and abstract screening was complete, the two screening authors reviewed any discrepancies until consensus was reached. The remaining included articles were then screened based on full text. Following the full text review, the two authors again reviewed any discrepancies to decide the final included articles. A third author was available if needed to support the two reviewing authors in coming to a consensus over whether an article should be included. Interrater reliability was recorded using Rayyan.

### Eligibility criteria

Studies were included if they applied a biological intervention to treat patients of all ages and genders with both eating disorder and psychological trauma. Biological treatments were defined as any intervention aimed at altering brain function or neurobiological processes through direct manipulation of brain circuitry or neurochemical activity. Studies needed to report on at least one outcome measure of the effect of the biological intervention on eating disorder or trauma symptoms post-intervention. Studies published in all years and in all languages were included. No restrictions were set on study design. Grey literature was included. Biological treatments for eating disorders are a highly under-researched area, therefore limited exclusion criteria were applied. However, studies only using psychological interventions, animal studies, secondary or tertiary research, and studies from non-peer reviewed journals were excluded.

### Data extraction

Once the included articles were finalized based on the eligibility criteria, authors EvB and IdV extracted data on the type, dose, and duration of the biological intervention, study design, sample size, participant age and demographic information, body mass index, eating disorder and psychological trauma outcome measures, (serious) adverse events, and treatment effects. In the event of missing data, the corresponding author of each included article was contacted.

### Quality assessment (risk of bias within and across studies)

Following the data extraction, risk of bias was assessed using the Risk of Bias in Non-randomized Studies - of Interventions tool (ROBINS-I; 76). The ROBINS-I tool was chosen because it assesses articles based on the risk of bias in estimates of effectiveness and safety in studies without comparison groups. The tool has seven domains including bias due to confounding, bias in selection of participants, bias in classification of interventions, bias due to missing data, bias in measurement of outcomes, and bias in selection of the reported results. The overall risk of bias is assessed by determining the highest risk of bias across domains. For example, if a study was judged to be at a “serious risk of bias” on one domain, the overall bias would be considered “serious”. Authors EvB and EvdB used the ROBINS-I tool to rate each report independently, and cross-referenced to scores to ensure consensus.

### Synthesis

Once the data from the included reports was extracted and the risk of bias was assessed, the findings were compared. Because high heterogeneity of biological treatment type and patient groups were expected *a priori*, no meta-analysis to compare findings was conducted.

## Results

**Figure 1:**
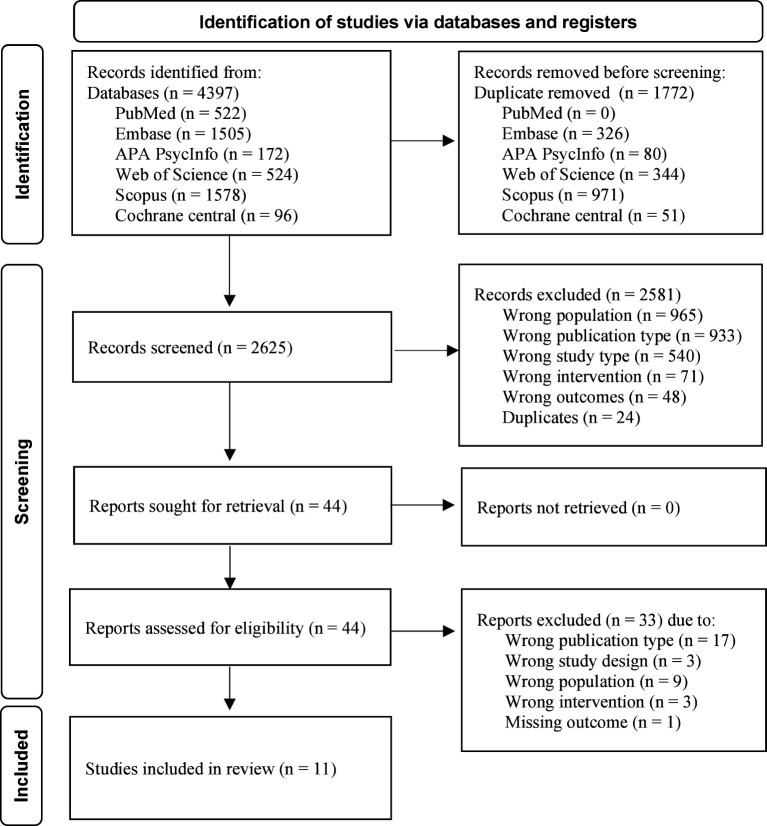
PRISMA flow diagram.

The search for biological treatments for eating disorders and PTSD yielded 2623 results, of which only 11 articles could be included. Please see **Figure 1** for the PRISMA flow chart of the identification of studies.

### Study characteristics

A total of 11 papers published between 1993 and 2023 were included in the review, with seven articles published in the last five years. The studies included were case reports (n = 4), prospective trials (n = 2), an open-label pilot (n = 1), a case series (n = 1), and randomized controlled trials (n = 3). Studies were conducted in the United States (n = 6), Canada (n = 3), Germany (n =1) and multinational (USA, Canada, and Israel; n = 1). A total of 142 adult female participants and six adult male participants were included in the studies, with a reported mean age of M = 32.5 (SD = 7.8). Among them, participants were diagnosed with anorexia nervosa (n = 59), bulimia nervosa (n = 57), binge eating disorder (n = 5), other specified feeding or eating disorder (n = 13), and night eating syndrome (n = 1). The studies reported on 88 participants with a diagnosis of PTSD and 21 with a trauma history without a reported diagnosis of PTSD. One study was conducted in an inpatient eating disorder treatment setting, while the other ten were carried out in an outpatient setting. A summary of the outcome measures and results is presented in **Table 1**.

**Table 1:** Characteristics of the included studies.

Authors and year	Intervention	Design	Sample Size	Sample & Sociodemographic characteristics Diagnosis, gender, age, race/ethnicity	Outcome measures	(Serious) Adverse Events	End-of-treatment effect	
							Eating Disorder	PTSD
Mahr et al., (2023) (77)	Prazosin Type: Alpha-1 antagonist Dose: 1 – 3 mg Frequency: daily	Pilot study	N = 8 Drop out after initial consent and screening: n = 2	DSM-IV Diagnosis: n = 8 Bulimia nervosa n = 8 PTSD Age: M = 30.12 (SD not reported) Sex: n = 8 female Body mass index: M = 22.75 (SD not reported) Race/Ethnicity/Nationality: Not Reported	PTSD: Sleep-50 Clinician-Administered PTSD Scale	Headache and nausea commonly reported One emergency room visit due to feeling faint	None reported	Prazosin decreased PTSD-related nightmare intensity compared to placebo
Winkeler et al., (2022) (78)	Test: Infra-Low Frequency Neurofeedback Dose: 12, 30-minute sessions Frequency: twice weekly over 6 weeks Placebo: “Media supported relaxation” Dose: 12, 30-minute sessions Frequency: twice weekly over 6 weeks Additional treatments: - Inpatient treatment program - 40 minute discussion about symptoms since last session	2-arm Randomized controlled trial	N = 36	ICD-10 Diagnosis: n = 15 Anorexia nervosa n = 6 Atypical anorexia nervosa n = 13 Bulimia nervosa n = 1 Atypical bulimia nervosa n = 1 Other eating disorders n = 36 PTSD Age: M = 28.4 (SD = 5.89) Sex: n = 36 female Body mass index: Test group: M = 18.22 (SD = 3.81) Placebo group: M = 22.59 (SD = 8.04) Race/Ethnicity/Nationality: Not Reported	Eating disorder: Eating Disorder Examination-Questionnaire Body mass index PTSD: Impact of Event Scale-Revised	Test group: None reported Placebo group: Non-compliance with weight agreement (n = 4) Severe self-injurious behaviour (n = 1)	Reduction in restrained eating behaviour in both groups Underweight participants in the test group had an increase in Body mass index compared to underweight participants in the placebo group that had no change in Body mass index	Test group showed less avoidance post-treatment compared to placebo group
Brewerton et al., (2022) (79)	Test: Oral MDMA Dose: 80–180 mg, followed by half-dose Frequency: Every 4 weeks Placebo: Oral placebo Frequency: Every 3-4 weeks Additional treatments: Therapist supported emotional resolution and finding meaning of trauma, encouraged welcoming and exploring difficult emotions, expressing, and understanding PTSD symptoms and their life impact	2-arm Randomized controlled trial	N = 90	DSM-V Diagnosis: N = 90 PTSD Unspecified diagnostic tool: n = 5 Binge eating disorder n = 9 Otherwise specified eating disorder Age: M = 41 (SD = 12.00) Sex: n = 58 female n = 31 male Body mass index: Test group: M = 26.0 (SD = 4.8) Placebo group: M = 24.8 (SD = 4.2) Ethnicity: n = 8 Hispanic or latino n = 80 Not hispanic or latino n = 1 Not reported Race: n = 3 American Indian or Alaska Native n = 7 Asian n = 2 Black or African American n = 68 White n = 7 More than one	Eating disorder: Eating Attitudes Test Body mass index PTSD: Clinician-Administered PTSD Scale	None mentioned Drop out: 7, reason not specified	Reduction in disordered eating attitudes in the test group compared to the placebo group Reduction in disordered eating attitudes in females with high baseline scores in the test group compared to the placebo group at follow-up No change in body mass index between the test group and the placebo group at follow up	None reported
Ragnhildstveit et al., (2021) (80)	Intravenous ketamine assisted psychotherapy. Dose: 0.5 mg/kilogram suspended in 0.9% normal saline Frequency: 6 sessions 2 times per week for 3 weeks (18 sessions total); repeated 3 times with one month in between. Additional treatments: 30 minutes of preparatory psychotherapy, person-centered, humanistic therapy during sessions intravenously over 40 min 20mEq Potassium chloride 100 mg tramadol	Case report	N = 1	DSM-V Diagnosis: Bulimia nervosa PTSD Age: 21 Sex: Female Body mass index: 17.6 Nationality: American Race/Ethnicity: Not reported	Eating disorder: Eating Disorder Examination-Questionnaire Tracking log for binge eating and purging episodes Patient perspective	Dissociation Ego dissolution, and perceptual distortions during all sessions Diplopia Nystagmus Alogia	Reduction on restraint, eating concern, shape concern and weight concern Reduction in total binge eating and purging episodes Patient reported a reduction in eating disorder thoughts and compulsions and improved body confidence	Improvement in PTSD symptoms "Unlocked" repressed memories from childhood, helped safely re-experience painful emotions Improvements in guilt, shame, and self-hatred (i.e., emotion dysregulation and personal beliefs)
Schwartz et al., (2021) (81)	Case 1: Intramuscular ketamine Dose: 0.4 mg/kilogram in right deltoid and 0.4 mg/kilogram in left deltoid Frequency: Injections 24 hours apart, every 3-5 weeks over 18 months Additional treatments: Outpatient treatment, weekly therapy and dietician appointments, monthly psychiatrist appointments 10mg Aripiprazole 40mg Fluoxetine	Case series	N = 1	DSM-V Diagnosis: Anorexia nervosa, restricting type PTSD Age: 49 Sex: female Body mass index: 19 Race: White Ethnicity/Nationality: Not reported	Eating disorder: Eating Disorder Examination Questionnaire (EDE-Q) Body mass index Patient perspective	Not specified between participants: 30-90 minute ketamine dissociative “trip” Post-treatment lethargy Headache	Reduction of weight and shape concern post-injection, without sustained effect (rebound in severity prior to follow-up treatment Patient reported a reduction in rigid and obsessive thoughts around food, and improved energy and motivation Increase in body mass index from 19 to 22.6	None reported
	Case 4: Intravenous ketamine, then intramuscular Dose: starting 0.5 mg/kilogram and progressively increasing. Maximum and maintained dose was 0.85 mg/kilogram Frequency: weekly injections Additional treatments: 150mg venlafaxine 200mg lamotrigine Topiramate (dosage not reported)		N=1	DSM-V Diagnosis: Anorexia nervosa, restrictive subtype Unspecified diagnostic tool: PTSD Age: 35 Sex: Female Body mass index: 13.5 Race/Ethnicity/Nationality: Not Reported	Eating disorder: Examination-Questionnaire Body mass index PTSD: Participant perspective		Slight improvements on restraint, eating concern, shape concern and weight concern Body mass index change from 37.8 to 39.2	Patient reported worsening stress and PTSD symptoms
Woodside et al., (2021) (82)	Intervention: Bilateral dorsomedial prefrontal cortex repetitive transcranial magnetic stimulation Dose: 10 Hertz stimulation Frequency: Initial course of 20 sessions, administered once daily on weekdays over 4 weeks Extension to 30 sessions offered for those with partial clinical improvement	Pilot trial	N = 19	DSM-IV Diagnosis N = 19 anorexia nervosa (no subtype reported) Unspecified diagnostic tool: n = unknown, PTSD Age: M = 31.2 (SD = 9.8) Sex: n = 19 Female BMI: M = 16.4 (SD = 1.3) Race/Ethnicity/Nationality: Not reported	Eating disorder: Eating Disorder Examination Questionnaire Body mass index	None mentioned	Reduction on total eating disorder severity, shape concern subscale, and weight concern subscale between pre- and post-treatment Body mass index decreased from 16.4 pre-treatment to 16.3 post-treatment	None reported
Pacilio et al., (2019) (83)	Intervention: Unilateral, right-sided electroconvulsive therapy Dose: 11 Ultra-brief pulse right unilateral treatments Frequency: 3 times per week Additional treatments: Methohexital, succinylcholine, and glycopyrrolate pretreatment for anesthesia Daily: paroxetine 40mg, aripiprazole 2.5mg, trazodone 50mg	Case report	N = 1	Diagnosis ( Unspecified diagnostic tools): Anorexia nervosa PTSD Age: 30 Sex: female Body mass index: 15.2 Ethnicity: African American Race and Nationality: Not reported	Patient perspective Body mass index	Worsening suicidal ideation less than 2-weeks post-treatment (patient reported this was due to social stressors)	Patient perspective: maintaining weight and food intake, still anxious about weight gain Body mass index increase from 15.2 to 15.7	None reported
Lipsman et al., (2017) (84)	Intervention: Deep Brain Stimulation Dose: Open-label continuous stimulation Frequency: For the entire 1-year study duration	Prospective open-label trial	N = 16	DSM-IV (text revision) Diagnosis: n = 7 Anorexia nervosa, restricting subtype n = 9 Anorexia nervosa, binge-eating/purging subtype Unspecified diagnostic tool: n = 10 PTSD Age: M = 34 (SD = 8) Sex: n =16 Female Body mass index (at surgery): M = 13.83 (SD = 1.49) Race/Ethnicity/Nationality: Not reported	Eating disorder: Body mass index Binging episodes Purging behaviour Yale-Brown-Cornell Eating Disorders Scale, preoccupation Yale-Brown-Cornell Eating Disorders Scale, rituals	Pancreatitis, hypokalaemia, refeeding delirium, hypophosphatraemia, worsening mood, QT prolongation, seizure, surgical-site infection, intraoperative pain, panic attack, increased lead impedance, fracture 2 participants requested the device be removed (no reason reported)	Body mass index increase from M=13.83 to M=17.34 No change in mean frequency of binging and purging. Reduction in eating preoccupations and eating disorder rituals Alterations in cerebral glucose metablism in anorexia-related brain circuits	None reported
Woodside et al., (2017) (85)	Repetitive transcranial magnetic stimulation in the dorsomedial prefrontal cortex Dose: 10 participants: 10 Hertz stimulation. 3 participants: Intermittent theta-burst stimulation, 120% resting motor threshold, 50 Hertz triplet bursts, 5 bursts per second, 2 on 8 off, 20 trains, 600 pulses per hemisphere, bilateral 1 participant: 20 Hertz stimulation, 120% resting motor threshold, 20 Hertz trains, 2.5 on 10 off, 30 trains, 1,500 pulses per hemisphere, bilateral Frequency: Initial course of 20 sessions, extended to 30 sessions in treatment responders	Case series	N=14	DSM-IV Diagnosis: n = 2 Anorexia nervosa, restrictive subtype n = 4 Anorexia nervosa, binge-purge subtype n = 5 Bulimia nervosa n = 3 Eating disorder not otherwise specified n = 14 Post Traumatic Stress Disorder Age: M = 39.8 (SD = 10.9) Sex: n = 14 female Body mass index: M = 20.81 (SD = 4.54) Race/Ethnicity/Nationality: Not reported	PTSD: PTSD Checklist – Civilian Version	Transient headaches during the first few sessions	None reported	Decrease in PTSD symptoms post-treatment
Tucker et al., (2004) (86)	Topiramate Dose: 25mg for one week, then titrated by 25 mg/week to 100 mg/day. After 3 months decreased to 75mg/day. After 2 weeks decreased to 50 mg/day. Frequency: daily Additional treatments: 10mg escitolapram for 10 days of the month 850mg metformin 2x daily 20mg simvastin daily 50mg hydrochlorothiazide 100mcg levothyroxine	Case study	N = 1	Unspecified diagnostic tool: Night-eating syndrome PTSD Age: 40 Sex female Body mass index: Not reported Race/Ethnicity/Nationality: Not reported	Eating disorder: Weight loss Episodes of sleepwalking plus binge eating Patient perspective PTSD: Patient perspective	Anomia	Reduction in frequency of binge eating while sleepwalking 70 lbs weight loss over 9 months (towards achieving a healthy weight)	Improved ability to discuss traumatic death of her daughter
McCarthy et al., (1993) (87)	Fluoxetine Dose: 60mg Frequency: daily	Single-blind, multicentre, placebo-controlled trial	N = 30	DSM-Third Edition, Revised Diagnosis: n = 30 Bulimia Nervosa n = 21 history of physical or sexual abuse Age: M = 28.6 (SD = 8.4) Sex: n= 29 female n = 1 male Body mass index: Not reported Race/Ethnicity/Nationality: Not reported	Eating disorder: Number of binges per day Physician Global impression scale Patient global impression scale	None mentioned Dropout: 2 participants	In the test group, there was no difference in the number of binge episodes between individuals with and without a history of physical abuse Main effect of physical abuse on global impression scale by the treating physician	None reported

M, Mean; SD, Standard Deviation; BMI, Body mass index; n, Number of participants; PTSD, PTSD; Mg, miligrams; Mcg, micrograms; DSM-IV/DSM-5, Diagnostic and Statistical Manual of Mental Disorders (8); Fourth/Fifth Edition; ICD-10, International Classification of Diseases; Tenth Edition; MDMA, 3,4-Methylenedioxymethamphetamine (commonly referred to as ecstasy).

### Biological treatments

Of the included studies, two categories of biological treatments were found: pharmacological interventions and neuromodulation. Six studies examined pharmacological interventions: three examined repurposed prescription medications, and three studies examined psychoactive substances with hallucinogenic and entactogenic properties. Repurposed prescription medications included one alpha-1 antagonist, one antidepressant, and one anticonvulsant. The studies examining hallucinogenic medicines and entactogens included one on 3,4-methylenedioxymethamphetamine (MDMA) and two on ketamine. Of the studies examining brain stimulation, two examined repetitive transcranial magnetic stimulation (rTMS), one examined electroconvulsive therapy, one examined deep brain stimulation, and one examined neurofeedback.

#### Pharmacological interventions

##### Repurposed medications

Mahr et al. (77) investigated the effect of prazosin on PTSD-related nightmares in eight female participants with bulimia nervosa. The study was a randomized, double-blind, placebo-controlled crossover pilot trial. Each participant received 1 – 3 mg of prazosin daily for a period of three weeks and a placebo daily for another period of three weeks, separated by a one-week washout period. The study reported that prazosin significantly reduced the intensity of nightmares compared to the placebo, yet it did not decrease the frequency of nightmares. Additionally, prazosin did not impact other sleep parameters. No outcomes on eating disorder symptoms were reported. Adverse events included an emergency room visit due to faintness, and several participants reported experiencing headaches and nausea. There were no dropouts reported in the study.

In a study by McCarthy et al. (87), the effect of childhood trauma on the response to 60 mg of fluoxetine was examined in thirty outpatient male and female participants with bulimia nervosa. The study was a placebo-controlled trial, where participants received either fluoxetine or a placebo daily. A significant main effect of physical abuse was found on the Global Impression Scale as rated by the psychiatrist, suggesting that participants with a history of physical abuse showed a greater reduction in bulimia severity in response to fluoxetine compared to those without such a history. However, no differences were observed in the number of binge episodes between those with a history with or without physical abuse. Adverse events were not mentioned in the article, however two participants dropped out due to personal reasons.

The article of Tucker et al. (86) presents a case report on a patient with PTSD and binge eating while sleepwalking, who was prescribed 100 mg of topiramate daily. The study reported a reduction in binge eating episodes while sleepwalking, yet it did not specify the exact frequency of the remaining binge eating episodes after treatment. The participant lost 70 pounds over nine months, although no pre- or post-weight, nor body mass index were reported. The researchers reported an improved ability to speak about the traumatic death of her daughter. Adverse effects including word-finding difficulties and memory problems, which may have been exacerbated by the concurrent use of metformin (850 mg taken twice daily). Following adjustments in the dosages of both topiramate and metformin, these side effects diminished over time.

##### Hallucinogenic medicines and entactogens

In the study conducted by Brewerton et al. (79), the efficacy of MDMA-assisted therapy was examined in a cohort of 90 participants with PTSD, 15 of which had a diagnosed eating disorder. The study used a two-arm randomized controlled trial design. Eating disorder symptoms were evaluated in the total group using the Eating Attitudes Test-26. The findings revealed that MDMA-assisted therapy led to a significant reduction in EAT-26 scores compared to the placebo group, with particularly pronounced effects observed in female participants with elevated baseline scores. Furthermore, there were no significant alterations in body mass index between the MDMA-assisted therapy and placebo groups. No outcomes on the impact of the treatment on PTSD symptoms were reported. Adverse events were not mentioned in the article; however, seven participants discontinued the study with unspecified reasons.

The following three case reports examine the effectiveness of 0.4 – 0.5 mg of ketamine per kilogram of body weight in patients with eating disorders and PTSD, who had not responded to prior conventional eating disorder treatments. Ragnhildstveit et al. (80) presented a case (Case 1) of a 21-year-old woman with bulimia nervosa and PTSD who received six sessions of ketamine-assisted therapy over three weeks. Schwartz et al. (81) reported on two cases; one (Case 2) on a 49-year-old woman with restrictive anorexia nervosa and PTSD who received ketamine every 4 – 5 weeks for 18 months, and another (Case 3) on a 35-year-old woman with a history of anorexia nervosa and PTSD who received ketamine weekly.

The results varied across these cases in terms of eating disorder symptoms. Case 1 achieved a complete remission of binge-eating and purging episodes after 3 months of ketamine-assisted therapy, and showed improvements in eating restraint, eating concern, shape concerns, and weight concerns. Case 2 experienced a reduction in weight and shape concerns post-injection, although this effect was not sustained, with a rebound in symptom severity before follow-up treatments. However, improvements were noted in reduced rigidity and obsessive thoughts around food, along with increased energy and motivation. The patient’s body mass index also increased from 19 to 22.6 kg/m2 during treatment. In contrast, Case 3 did not demonstrate any substantial improvement in eating disorder symptoms. The first two cases did not report on any PTSD improvement, whereas case 3 noted worsening PTSD symptoms, attributable to new life stressors. Adverse events for Case 1 included dissociation, ego dissolution, and perceptual distortions during all ketamine sessions, along with diplopia, nystagmus, and alogia, all of which resolved after each session. Case 2 reported mild sedation, and no adverse events were reported in Case 3.

#### Neuromodulation

##### Non-invasive brain stimulation

Three studies reported on non-invasive brain stimulation: two on rTMS and one on electroconvulsive therapy. The two studies examining rTMS targeted the dorsomedial prefrontal cortex in 14 patients with various eating disorders (85) and 19 with anorexia (82), all with comorbid PTSD. Both studies used between 10 - 20 Hz over 20 to 30 sessions. In the 2017 study, all participants showed improvements in PTSD severity after treatment using the PTSD Checklist. Of note, two participants with a body mass index under 15.7 kg/m2 had smaller improvements in PTSD severity compared to participants with a higher body mass index. No eating disorder outcomes were reported. In contrast, the 2021 study noted improvements in global eating disorder severity, along with significant improvements in shape concern and weight concern post-treatment. However, rather than improving, the body mass index deteriorated slightly from an average of 16.4 kg/m2 pre-treatment to 16.3 kg/m2 post-treatment. No trauma outcomes were reported. It is challenging to compare outcomes between these studies because the 2017 study on patients with various eating disorders only reported a reduction in PTSD symptoms while the 2021 study on patients with anorexia only reported on reductions on eating disorder severity. As far as adverse events, the 2017 study noted headaches during the first few treatment sessions, while the 2021 study did not mention any adverse events.

The study by Pacilio et al. (83) investigates the efficacy of electroconvulsive therapy through a case report of a 30-year-old woman with anorexia nervosa and PTSD. Due to insufficient response to conventional eating disorder treatments, she underwent eleven electroconvulsive therapy sessions. Over the course of the intervention, her body mass index increased from 15.2 to 15.7 kg/m2, however she reported experiencing guilt and anxiety around eating and weight gain. No improvement in PTSD symptoms was reported during or after the electroconvulsive therapy sessions. No adverse events during the electroconvulsive therapy were reported, however following discharge, the patient experienced a relapse of suicidal thoughts which she attributed to persistent psychosocial stressors, including homelessness.

##### Invasive brain stimulation

One prospective open-label trial examined the efficacy of deep brain stimulation in 16 patients with chronic and treatment-resistant anorexia nervosa, ten of whom had post-traumatic stress disorder (84). The researchers found that the mean body mass index increased from 13.8 to 17.3 kg/m2 after 12-months. Notably, three of the eight patients with binge eating episodes achieved remission. Additionally, patients exhibited significant improvements in eating disorder-related psychopathology, including eating-related preoccupations and rituals associated with the eating disorder, as measured by the Yale-Brown Cornell Eating Disorder Scale. No PTSD outcomes were reported. One patient developed a surgical-site infection that required device removal after initially responding to antibiotics. Two patients experienced seizures, one potentially linked to hyponatremia, and one with no identifiable cause. One patient requested her stimulator be deactivated after nine months due to discomfort related to recovering from an underweight state, while another withdrew from the study after six months for reasons which the authors describe as “unclear”. Further adverse events included five patients experiencing prolonged incision site pain lasting beyond the typical recovery period of 3–4 days, and hyponatremia and hypokalemia which may have been attributable to the underlying eating disorder.

##### Neurofeedback

The study by Winkeler et al. (78) investigated the effectiveness of infra-low frequency neurofeedback as an adjunctive treatment for patients with eating disorders and PTSD in a randomized placebo-controlled trial involving a total of 36 female participants. Participants included those with anorexia nervosa, bulimia nervosa, and other specified eating disorders. The results suggest that both the intervention and control groups exhibited improvements in restraint eating, with the neurofeedback group showing greater enhancement compared to the placebo group. Both groups demonstrated reductions in concerns related to eating and body shape; however, the differences between the intervention and placebo groups were less pronounced in this regard. Notably, the body mass index of participants in the underweight neurofeedback group increased significantly more than that in the placebo group.

Additionally, both groups experienced a significant decrease in hyperarousal; however, the reduction was not significantly different between neurofeedback and placebo groups. The neurofeedback group also showed a significant reduction in avoidance of cognitive or behavioral contact with the traumatic situation while the control group did not exhibit any change. Within the neurofeedback group no adverse events were reported. In contrast, the placebo group experienced one case of severe self-injurious behavior (n = 1) and four cases of non-compliance with the weight agreement (n = 4).

### Quality assessment

The quality assessment for the present study was done using the Risk of Bias in Non-randomised Studies-of Interventions (ROBINS – I, 76) tool, as it was expected that most included studies would be non-randomized controlled studies. The exploratory nature of many of the included studies led to a high risk of bias across studies. Of the eleven studies included in this review, eight were considered having a serious risk of bias, one was considered at a moderate risk of bias, and one was considered at a low risk of bias. Across the pre-intervention and at-intervention domains, bias due to confounding across the studies was determined to be serious (n = 4), moderate (n = 3), and low (n = 5). An example of confounding was having multiple concurrent interventions, such as combining brain stimulation with psychiatric medications or psychotherapy. Bias in selection of participants into the study was serious (n = 5), moderate (n = 2), and low (n = 4), such that most studies did not report how participants were selected. Bias in the classification of interventions across all studies was low (n = 11), as all studies defined the investigational intervention clearly in terms of type, dose and frequency. As far as the post-intervention domains, bias due to deviations from intended interventions was mostly low (n = 9), although one study had a moderate bias (n = 1), and one had a serious bias due to deviations from the intended protocol (n = 1). Serious bias due to missing data was frequent (n = 5), as many studies reported missing baseline or outcome data. Three studies had a moderate risk of bias due to missing data (n = 3) and three had a low risk of bias due to missing data (n = 3). Bias in the measurement of outcomes was frequently serious (n = 9) or moderate (n = 2), with one study having a low risk bias due to outcomes (n = 1). Lastly, bias in the selection of the reported result was frequently serious (n = 9), followed by moderate (n = 2) and low (n = 1).

The ROBINS-I criterion measure takes the highest risk of bias across all domains, and in this review most studies (n = 10) were considered at serious risk of bias, with only one study at a moderate risk of bias (n = 1) and one at a low risk of bias (n = 1). Because this review aims to examine a highly under-researched area, and no studies were considered as having a critical risk of bias, no studies were excluded based on poor quality. See Annex B for the complete ROBINS-I quality assessment.

## Discussion

This systematic review aimed to gather and synthesize all published research on biological treatments for patients with eating disorders and trauma symptoms, determine the safety and efficacy of these interventions, and identify areas for future research. Despite an exhaustive worldwide search of the current literature, only eleven articles in the past 31 years have evaluated biological treatments to support those with eating disorders and PTSD. This is particularly surprising as an estimated 55.5 million people worldwide have an eating disorder (88). Assuming the conservative findings that 18% of eating disorder patients having co-occurring PTSD, nearly 10 million people worldwide are estimated to have both disorders.

The studies examining biological treatments in this review included two articles on ketamine, two on rTMS, and the other seven were on distinct interventions. All the biological treatments included in this review reported on some positive effects on trauma and/or eating disorder symptoms.

The three patients who received ketamine (80, 81) reported decreases in eating disorder pathology using the Eating Disorder Examination, which is considered the “gold-standard” measure of changes in eating disorder pathology. While the eating disorder improvements are promising, changes in PTSD symptoms varied across patients; in the case report by Ragnhildstveit et al. (80), ketamine had a positive effect on the patient’s PTSD symptoms, as it helped “unlock” repressed memories, safely re-experience emotions, and decrease symptoms of feeling of guilt, shame, and safe-hatred relating to the trauma. In the study by Schwartz et al. (81), no PTSD symptoms were reported for one patient, and the second patient experienced worsening PTSD symptoms. One reason for the variation in ketamine effect found in this review could be the frequency of ketamine injection. In the study by Ragnhildstveit et al. (80), the patient had 18 sessions within three weeks, whereas in the study by Schwartz et al. (81), the patient with no PTSD outcomes reported received an injection every day, while the patient with worsening PTSD symptoms only had one ketamine injection weekly. Interestingly, all three patients included in this review had received previous eating disorder treatments with no effect, suggesting ketamine may be worth exploring further as an adjunct therapy or in a stepped-care model for patients who have experienced more eating disorder chronicity or resistance to earlier lines of treatment.

Prior work examining ketamine’s effect in treating PTSD have also found mixed findings; some studies have found ketamine was effective for treating chronic PTSD (89), while others have found that ketamine exacerbated symptoms of PTSD including reexperiencing and hyperarousal (90, 91). While dosage and frequency may be important, another factor worth considering is the duration of PTSD symptoms. A systematic review by Du et al. (92) found that while ketamine was effective for those with chronic PTSD, it exacerbated symptoms in those with more acute PTSD. One reason for this could be ketamine’s significant role in glutamate transmission and promotion of synaptic plasticity in the prefrontal cortex, which can rapidly improve psychiatric symptoms including depression (93, 94). However, for those experiencing acute trauma symptoms, ketamine may overstimulate the glutamate-glucocorticoid system, leading to fragmented memory formation and dissociation (95).

Of the two studies examining rTMS in 33 patients, one study only reported on eating disorder outcomes (82) and the other study only reported on trauma outcomes (85), however both studies found positive effects on eating disorder pathology and PTSD symptoms, respectively. While Woodside et al. (82) examined the effect on eating disorder pathology in patients with comorbid anorexia and PTSD, prior work has found promising effects of rTMS across patients with eating disorder diagnoses; overweight patients with obesity and binge eating disorder have shown decreases in body mass index and decreased urges to eat (96, 97), patients with bulimia have shown decreases in vomiting and laxative/diuretic use (98), and patients with anorexia have shown reduced body image concern and improved attitudes towards food (99). While Woodside et al. (85) aimed to improve PTSD symptoms by targeting the dorsomedial prefrontal cortex, a systematic review and meta-analysis examining rTMS to the adjacent dorsolateral prefrontal cortex found the stimulation to be both safe and effective in reducing PTSD pathology compared to the sham condition (100).

The two other studies examining brain stimulation through electroconvulsive therapy and deep brain stimulation targeted regions beyond the prefrontal cortex, suggesting both eating disorders and PTSD have broader and more complex functional networks. Despite the study by Pacilio et al. (83) including a literature review on electroconvulsive therapy for eating disorders, the researchers did not report a rationale for offering right-sided unilateral stimulation. As far as eating disorder and PTSD symptoms, the patient’s benefits were limited, with a marginal increase in body mass index (despite still being severely underweight) and a reported increase in her dietary intake. In contrast, the study examining deep brain stimulation in the subcallosal cingulate for patients with anorexia (84) found significant improvements in body mass index and changes in anorexia-related brain circuits.

Although the study examining infra-low frequency neurofeedback did not directly aim to stimulate any specific brain regions, the researchers highlight the benefits of neurofeedback in increasing neural network connectivity and discuss how improvements in connectivity may support improved stimuli integration in patients with PTSD (78, 101).

The current understanding of the effects of various forms of brain stimulation for both eating disorders and PTSD is rapidly evolving. All forms of brain stimulation covered in this review, including rTMS, deep brain stimulation, and electroconvulsive therapy, aim to target specific neural circuits which may be disrupted in eating disorders and/or PTSD. In particular, many studies have targeted the dorsomedial prefrontal cortex using rTMS, as this area is responsible for emotion regulation and cognitive control, and it may have decreased connectivity in those with eating disorders and PTSD (82, 102, 103). Muratore and Attia (104) compare and summarize the scientific literature on rTMS for anorexia, targeting the left dorsolateral prefrontal cortex, and across studies it has been found effective in decreasing eating disorder symptoms including self-reported urges to restrict, feeling fat, and feeling full and increasing BMI. Similarly, a systematic review on rTMS for PTSD found that all ten studies included studies targeted the dorsolateral prefrontal cortex and provided evidence that rTMS may be an effective treatment for PTSD by improving emotion and fear regulation through inhibition of fear circuits in the amygdala (105).

The three independent studies examining the repurposed medications topiramate, prazosin, and fluoxetine all reported some improvements in either PTSD or eating disorder symptoms. The case study on topiramate found the patient had a reduction in nighttime binge eating and was able to begin discussing the traumatic death of her child (86). While topiramate is indicated as an anticonvulsant medication, a meta-analysis by Nourredine et al. (106) has found it effective in reducing binge-eating episodes in patients with binge eating disorder, and prior work has demonstrated topiramate’s efficacy in reducing PTSD symptoms (107). Interestingly, topiramate inhibits glutamatergic excitation by blocking AMPA and glutamate receptors (108), which has been shown to decrease binge-eating episodes in those with bulimia nervosa and binge-eating disorder and support weight gain in those with anorexia nervosa (48). These findings together highlight the role of glutamatergic transmission in both eating disorder and PTSD symptom reduction, and the importance of researching this area further.

Similarly, while prazosin is an alpha-1 antagonists indicated for hypertension, a literature review and meta-analysis have both found it effective in improving hyperarousal, decreasing nightmares, and generally improving sleep-related disturbances in patients with PTSD (109, 110), further endorsing the findings included in this review (77). Lastly, the study by McCarthy et al. (87) included in this review examined fluoxetine for patients with bulimia and childhood trauma and concluded that it was effective in decreasing dissociative episodes and binge eating. Although fluoxetine has been extensively researched as a treatment for depression, very little work has supported its efficacy in PTSD. Fluoxetine primarily acts as a selective serotonin reuptake inhibitor; however, it can also increase Allo, a neurosteroid which is often low in those with PTSD (111). Increases and normalization of Allo levels may support improved anxiety, fear, and aggression in those with PTSD (112). Although a randomized controlled trial found fluoxetine decreased clinician-rated PTSD symptom severity (113), a follow-up randomized controlled trial comparing fluoxetine, eye movement desensitization and reprocessing, and placebo, found that fluoxetine was no better than placebo (114).

Although evidence for the effect of repurposed medications in this group is conflicting, one pharmacological intervention that offers promise is MDMA. The study included in this review found significant improvements in eating disorder attitudes in a group of patients with PTSD, however no PTSD outcomes were reported (79). Despite the lack of PTSD outcome reporting in this study, the evidence and support for MDMA-assisted psychotherapy for PTSD is rapidly increasing. MDMA was designated a “breakthrough therapy” for PTSD in 2017 by the United States Food and Drug Administration, which has expedited the drug development pathways and supported multiple phase II trials for PTSD (115). MDMA-assisted therapy may be particularly effective for patients who have treatment-resistant PTSD or those who struggle to recognize and respond to their emotions (116, 117). MDMA has a significant effect on serotonin, norepinephrine, and dopamine circuits, which can lead to increased emotion regulation, distress tolerance, and feelings of reward (115). MDMA may also support developing a greater window of tolerance and the promotion of fear extinction, which are two critical aspects of PTSD treatment (115). MDMA’s use in eating disorder populations is somewhat controversial; although increased emotion regulation and a greater window of tolerance may support patients who are struggling with symptoms such as restrictive eating or binge-eating, MDMA can also cause a lack of appetite or vomiting (118), which can be unsafe for those with eating disorders.

The findings from this review align with the current literature suggesting that the dorsolateral cortex, dorsomedial prefrontal cortex, and subcallosal cingulate may all be implicated in eating disorders and PTSD and should be explored further. Both fMRI and intrinsic functional connectivity analyses demonstrate that patients with PTSD may have decreased connectivity between these regions (68, 69) which can disrupt mood regulation, emotional processing, and impulse control (69). Together these findings highlight the importance of researching the functional connectivity in eating disorders and PTSD and effectively targeting these areas through biological treatments.

Seven of the eleven studies were at a serious risk of bias based on the ROBINS-I assessment. However, due to the exploratory nature of biological treatments for those with co-occurring eating disorders and psychological trauma, a high risk of bias was anticipated. It is important to acknowledge that although some studies were categorized as having a serious risk of bias based on the ROBINS-I criteria, their contribution to this under-researched field remain significant. Most of the studies included in this review were exploratory in nature and have provided the first steps in developing effective treatments for this complex comorbidity. The small sample sizes and reliance on researchers’ impressions and patients’ self-report make it difficult to interpret the effectiveness of any single intervention. Furthermore, only four studies reported on both eating disorder and trauma outcomes, limiting the generalizability of the findings. Promisingly, all biological treatments included in this review had some improvement in either eating disorder or post-traumatic stress disorder symptoms in those with co-occurring eating disorders and psychological trauma, underscoring the need for follow-up studies using validated outcome measures with larger sample sizes. While many of the findings are preliminary, these studies together highlight the importance of further exploring the clinical effectiveness of biological intervention for those with co-occurring eating disorders and PTSD. Given the aim of this study was to examine the clinical use and safety of biological treatments for eating disorders and trauma, pre-clinical data was not included. Given the heterogeneity of the included studies, follow-up studies should aim to incorporate pre-clinical data to provide a full overview of biological treatments for eating disorders and PTSD.

## Conclusion

The promising early findings from this review highlight the importance of future work in evaluating the efficacy of biological interventions for those with PTSD and eating disorders. Several recommendations for future work in this area are as follows: firstly, studies should aim to use validated PTSD and eating disorder outcome measures. The use of validated measures, such as the eating disorder examination (EDE; 15), or the Clinician-Administered PTSD Scale for DSM-5 (CAPS-5) will support a better understanding of the effectiveness of each intervention, and allow for a better comparison between intervention. Secondly, future work should aim to include physiological outcome measures, including neuroimaging, changes in drug serum concentration, and other biomarkers. Including physiological changes and biomarkers will support a broader understanding of each intervention’s mechanism of action and provide evidence for or against current theories in brain circuitry and stress responses.

This systematic review offers the first steps in determining what biological treatments are available for patients with eating disorders and PTSD, which have preliminary evidence for safety and efficacy, and which warrant future research. Biological treatments offer a promising route to improving treatment and outcomes for those with co-occurring eating disorders and psychological trauma, as many experience illness chronicity and relapse. The findings from the present study also emphasize the strong biological and neurological underpinnings of both eating disorders and psychological trauma and support the isolation and targeting of key neural circuits to support recovery. The present study is the first to systematic review and synthesize all existing research into biological treatments for those with eating disorders and psychological trauma. Together, the findings preliminarily suggest that various biological interventions may be safe and effective for those with co-occurring eating disorders and psychological trauma, and researchers and clinicians should aim to explore their effectiveness through larger-scale studies using validated outcome measures.

## Data Availability

The original contributions presented in the study are included in the article/**Supplementary Material**. Further inquiries can be directed to the corresponding author.
