# Supplementary material for: Biological treatments for co-occurring eating disorders and psychological trauma: a systematic review
Source: Front Psychiatry. 2025 Feb 21;16:1523269. doi: 10.3389/fpsyt.2025.1523269 (PMC11914888; doi:10.3389/fpsyt.2025.1523269)
Supplement: Supplementary file 1 [file DataSheet1.docx]

**ANNEX A: SEARCH STRING**

# Databases

PubMed 🡪 medical database (free)

Embase 🡪 medical database (paid) no conference abstracts/ preprints

PsycInfo ( psychological database

Web of Science 🡪 all sciences

Scopus 🡪 all sciences

Cochrane 🡪 evidence based SR + trial database

Elements of the search query – building block method/PICO:

**# Eating and feeding disorders**

"Feeding and Eating Disorders"[Mesh] OR “eating disorder*”[tiab] OR “feeding disorder*”[tiab] OR “binge eating*”[tiab] OR “bulimic*”[tiab] OR “bulimia”[tiab] OR “boulimic*”[tiab] OR “anorexi*”[tiab] OR “anorectic”[tiab] OR “underweight*”[tiab] OR “Avoidant Restrictive Food Intake Disorder*”[tiab] OR “Diabulimi*”[tiab] OR “diaboulimi*”[tiab]

OR “Food Addiction*”[tiab] OR “Night Eating Syndrome*”[tiab] OR “Orthorexi*”[tiab] OR “Relative Energy Deficienc*”[tiab] OR “Female Athlete Triad Syndrom*” OR “self induced vomit*”[tiab] OR “purging behavio*”[tiab] OR “purging disorder*”[tiab] OR OSFED[tiab] OR EDNOS[tiab] OR ARFID[tiab]

**# PTSD – trauma**

"Stress Disorders, Traumatic"[Mesh] OR "Psychological Distress"[Mesh] OR “complex trauma*”[tiab] OR “posttraumatic stress*”[tiab] OR “post-traumatic stress*”[tiab] OR “posttraumatic-disorder*”[tiab] OR “post-traumatic disorder*”[tiab] OR "post traumatic neuros*"[tiab] OR "posttraumatic neuros*"[tiab] OR “traumatic distress*”[tiab] OR "posttraumatic psychos*"[tiab] OR "post-traumatic psychos*"[tiab] OR "posttraumatic syndrom*"[tiab] OR "post-traumatic syndrom*"[tiab] OR "trauma-and-stressor-related-disorder*"[tiab] OR “traumatic stress disorder*”[tiab] OR ptsd*[tiab] OR ptss*[tiab] OR “combat-disorder*”[tiab] OR “combat-fatigue*”[tiab] OR “combat-stress*”[tiab] OR “shell-shock*”[tiab] OR “combat-neuros*”[tiab] OR “war-neuros*”[tiab] OR “battle-fatigue*”[tiab] OR “psychological stress*”[tiab] OR “psychological trauma*”[tiab] OR psychotrauma*[tiab] OR “psycho-trauma*”[tiab] OR “trauma-focused*”[tiab] OR “psychological distress*”[tiab] OR “emotional trauma*”[tiab] OR “emotional distress*”[tiab] OR “emotional damag*”[tiab] OR “emotional injur*”[tiab] OR “mental damag*”[tiab] OR “mental harm*”[tiab] OR “mental injur*”[tiab] OR “mental trauma*”[tiab] OR “sexual trauma*”[tiab] OR “psychological damag*”[tiab] OR “psychological harm*”[tiab] OR “psychological injur*”[tiab] OR “Posttraumatische Belastungsstörung”[tiab] OR PTBS[tiab]

**# Childhood maltreatment – sexual abuse**

"Adult Survivors of Child Adverse Events"[Mesh] OR "Battered Child Syndrome"[Mesh] OR "Child Abuse"[Mesh] OR "Adverse Childhood Experiences"[Mesh] OR "Emotional Abuse"[Mesh] OR “Incest”[Mesh] OR "Sex Offenses"[Mesh] OR "Intimate Partner Violence"[Mesh] OR "Violence"[Mesh] OR “childhood trauma*”[tiab] OR “childhood adverse event*”[tiab] OR “childhood adversit*”[tiab] OR “adverse childhood experien*”[tiab] OR “battered child*”[tiab] OR “child abus*”[tiab] OR “child sexual abus*”[tiab] OR “childhood sexual abus*”[tiab] OR “child maltreatment*”[tiab] OR “childhood maltreatment*”[tiab] OR “child mistreatment*”[tiab] OR “child neglect*”[tiab] OR “childhood neglect*”[tiab] OR “childhood psychological trauma*”[tiab] OR “childhood emotional trauma*” [tiab] OR “childhood abus*”[tiab] OR (ACEs [tiab] AND child [tiab]) OR (ACE[tiab] AND child[tiab]) OR “child physical abus*”[tiab] OR “childhood physical abus*”[tiab] OR “early life adversit*”[tiab] OR “Incest*”[tiab] OR “sex offense*”[tiab] OR “sexual abus*”[tiab] OR “physical abus*”[tiab] OR “intimate partner violen*”[tiab] OR “spouse abus*”[tiab] OR rape*[tiab]

Extra Mesh-termen?

"Sex Offenses"[Mesh]

[Anthropology, Education, Sociology and Social Phenomena Category](https://www.ncbi.nlm.nih.gov/mesh/1000073)

[Social Sciences](https://www.ncbi.nlm.nih.gov/mesh/68012942)

[Criminology](https://www.ncbi.nlm.nih.gov/mesh/68003418)

[Crime](https://www.ncbi.nlm.nih.gov/mesh/68003415)

Sex Offenses

[Child Abuse, Sexual](https://www.ncbi.nlm.nih.gov/mesh/68002650)

[Human Trafficking](https://www.ncbi.nlm.nih.gov/mesh/68064410)

[Rape](https://www.ncbi.nlm.nih.gov/mesh/68011902)

[Anthropology, Education, Sociology and Social Phenomena Category](https://www.ncbi.nlm.nih.gov/mesh/1000073)

` [Social Sciences](https://www.ncbi.nlm.nih.gov/mesh/68012942)

[Criminology](https://www.ncbi.nlm.nih.gov/mesh/68003418)

[Crime](https://www.ncbi.nlm.nih.gov/mesh/68003415)

[Violence](https://www.ncbi.nlm.nih.gov/mesh/68014754)

[Domestic Violence](https://www.ncbi.nlm.nih.gov/mesh/68017579)

[Child Abuse](https://www.ncbi.nlm.nih.gov/mesh/68002649)

**Child Abuse, Sexual**

[Psychiatry and Psychology Category](https://www.ncbi.nlm.nih.gov/mesh/1000070)

[Mental Disorders](https://www.ncbi.nlm.nih.gov/mesh/68001523)

[Trauma and Stressor Related Disorders](https://www.ncbi.nlm.nih.gov/mesh/2009790)

[Stress Disorders, Traumatic](https://www.ncbi.nlm.nih.gov/mesh/68040921)

**Sexual Trauma**

[Military Sexual Trauma](https://www.ncbi.nlm.nih.gov/mesh/2103375)

**#** **Non-psychological treatment**

"Antidepressive Agents"[Mesh] OR "Antidepressive Agents" [Pharmacological Action] OR "Anti-Anxiety Agents"[Mesh] OR "Anti-Anxiety Agents" [Pharmacological Action] OR "Tranquilizing Agents"[Mesh] OR "Psychotropic Drugs"[Mesh] OR "Antipsychotic Agents"[Mesh] OR "Antipsychotic Agents" [Pharmacological Action] OR "Drug Therapy"[Mesh] OR "Off-Label Use"[Mesh] OR "Drug Repositioning"[Mesh] OR "Therapies, Investigational"[Mesh] OR "Deep Brain Stimulation"[Mesh] OR "Transcranial Magnetic Stimulation"[Mesh] OR "Transcranial Direct Current Stimulation"[Mesh] OR "Neurofeedback"[Mesh] OR "Hormone Replacement Therapy"[Mesh] OR "Hydrocortisone"[Mesh] OR "Oxytocin"[Mesh] OR "Epinephrine"[Mesh] OR "Norepinephrine"[Mesh] OR "Androgens"[Mesh] OR "Androgens" [Pharmacological Action] OR "Estrogens"[Mesh] OR "Estrogens" [Pharmacological Action] OR "Melatonin"[Mesh] OR "Leptin"[Mesh] OR "Ghrelin"[Mesh] OR "Exercise Movement Techniques"[Mesh] OR "Phenethylamines"[Mesh] OR "Mind-Body Therapies"[Mesh] OR "Complementary Therapies"[Mesh] OR "Acupuncture Therapy"[Mesh] OR "Phytotherapy"[Mesh] OR "Homeopathy"[Mesh] OR "Medicine, Traditional"[Mesh] OR "Holistic Health"[Mesh] OR "General Surgery"[Mesh] OR "Surgical Procedures, Operative"[Mesh] OR "Bariatric Surgery"[Mesh] OR "Electroconvulsive Therapy"[Mesh] OR "Magnetic Field Therapy"[Mesh] OR "Hyperbaric Oxygenation"[Mesh] OR "Life Style"[Mesh] OR "Dietary Supplements"[Mesh] OR "dietary supplement, SPORT" [Supplementary Concept] OR "Nutritional Support"[Mesh] OR Medication*[tiab] OR Antidepressant*[tiab] OR “anti-depressant*”[tiab] OR “Anti-anxiety agent*”[tiab] OR Antipsychotic*[tiab] OR “Mood stabilizer*”[tiab] OR tranquilizer*[tiab] OR Pharmacotherap*[tiab] OR “Pharmacological Intervention*”[tiab] OR “Psychotropic drug*”[tiab] OR “Off-label*”[tiab] OR repurposing[tiab] OR “Experimental drug*”[tiab] OR “Biological Intervention*”[tiab] OR “Deep brain stimulation”[tiab] OR “Transcranial magnetic stimulation”[tiab] OR TMS[tiab] OR “Transcranial direct current stimulation”[tiab] OR tDCS[tiab] OR Neurofeedback[tiab] OR “Endocrine Intervention*”[tiab] OR “Hormone therap*”[tiab] OR “Thyroid intervention*”[tiab] OR “Endocrine modulator*”[tiab] OR "Oxytocin"[tiab] OR "Epinephrine"[tiab] OR "Norepinephrine"[tiab] OR Insulin[tiab] OR Cortisol[tiab] OR Androgen[tiab] OR Estrogen[tiab] OR Melatonin[tiab] OR Leptin[tiab] OR Ghrelin[tiab] OR ”Lifestyle intervention*”[tiab] OR “Exercise therap*”[tiab] OR Yoga[tiab] OR “Tai chi”[tiab] OR “Tai Ji”[tiab] OR “Dance therap*”[tiab] OR “Substance-assisted therap*”[tiab] OR “MDMA-assisted therap*”[tiab] OR “Phenethylamin*”[tiab] OR “Amphetamin*”[tiab] OR “Alternative Complementary Therapies”[tiab:~3] OR Acupuncture[tiab] OR “Herbal remedie*”[tiab] OR Homeopath*[tiab] OR “traditional medicine”[tiab] OR “Holistic health intervention*”[tiab] OR “Medical Intervention*”[tiab] OR Surgery[tiab] OR “Electroconvulsive therap*”[tiab] OR ECT[tiab] OR “Electromagnetic therap*”[tiab] OR “Repetitive transcranial magnetic stimulation*”[tiab] OR rTMS[tiab] OR “Hypobaric oxygen therapy”[tiab:~3] OR “Sleep intervention*”[tiab] OR “Stress management”[tiab] OR “Dietary chang*”[tiab] OR “Nutritional intervention*”[tiab] OR “Dietary supplement*”[tiab] OR “Nutritional counsel*”[tiab] OR “Dietary intervention*”[tiab] OR “Nutrient supplementation*”[tiab] OR “Nutraceutical intervention*”[tiab]

# Meeting 1-3-2024

| **Search** | **Query** | **Results** |
| --- | --- | --- |
| #12 | Search: **#7 AND #11** | [2,369](https://pubmed.ncbi.nlm.nih.gov/?term=%237+AND+%2311&sort=relevance) |
| #11 | Search: **#9 OR #10** | [171,931](https://pubmed.ncbi.nlm.nih.gov/?term=%239+OR+%2310&sort=relevance) |
| #10 | Search: **"Adult Survivors of Child Adverse Events"[Mesh] OR "Battered Child Syndrome"[Mesh] OR "Child Abuse"[Mesh] OR "Adverse Childhood Experiences"[Mesh] OR "Emotional Abuse"[Mesh] OR "Incest"[Mesh] OR "childhood trauma*"[tiab] OR "childhood adverse event*"[tiab] OR "childhood adversit*"[tiab] OR "adverse childhood experien*"[tiab] OR "battered child*"[tiab] OR "child abus*"[tiab] OR "child sexual abus*"[tiab] OR "childhood sexual abus*"[tiab] OR "child maltreatment*"[tiab] OR "childhood maltreatment*"[tiab] OR "child mistreatment*"[tiab] OR "child neglect*"[tiab] OR "childhood neglect*"[tiab] OR "childhood psychological trauma*"[tiab] OR "childhood emotional trauma*" [tiab] OR "childhood abus*"[tiab] OR (ACEs [tiab] AND child [tiab]) OR (ACE[tiab] AND child[tiab]) OR "child physical abus*"[tiab] OR "childhood physical abus*"[tiab] OR "early life adversit*"[tiab] OR "Incest*"[tiab]** | [56,181](https://pubmed.ncbi.nlm.nih.gov/?term=%22Adult+Survivors+of+Child+Adverse+Events%22%5BMesh%5D+OR+%22Battered+Child+Syndrome%22%5BMesh%5D+OR+%22Child+Abuse%22%5BMesh%5D+OR+%22Adverse+Childhood+Experiences%22%5BMesh%5D+OR+%22Emotional+Abuse%22%5BMesh%5D+OR+%E2%80%9CIncest%E2%80%9D%5BMesh%5D+OR+%E2%80%9Cchildhood+trauma%2A%E2%80%9D%5Btiab%5D+OR+%E2%80%9Cchildhood+adverse+event%2A%E2%80%9D%5Btiab%5D+OR+%E2%80%9Cchildhood+adversit%2A%E2%80%9D%5Btiab%5D+OR+%E2%80%9Cadverse+childhood+experien%2A%E2%80%9D%5Btiab%5D+OR+%E2%80%9Cbattered+child%2A%E2%80%9D%5Btiab%5D+OR+%E2%80%9Cchild+abus%2A%E2%80%9D%5Btiab%5D+OR+%E2%80%9Cchild+sexual+abus%2A%E2%80%9D%5Btiab%5D+OR+%E2%80%9Cchildhood+sexual+abus%2A%E2%80%9D%5Btiab%5D+OR+%E2%80%9Cchild+maltreatment%2A%E2%80%9D%5Btiab%5D+OR+%E2%80%9Cchildhood+maltreatment%2A%E2%80%9D%5Btiab%5D+OR+%E2%80%9Cchild+mistreatment%2A%E2%80%9D%5Btiab%5D+OR+%E2%80%9Cchild+neglect%2A%E2%80%9D%5Btiab%5D+OR+%E2%80%9Cchildhood+neglect%2A%E2%80%9D%5Btiab%5D+OR+%E2%80%9Cchildhood+psychological+trauma%2A%E2%80%9D%5Btiab%5D+OR+%E2%80%9Cchildhood+emotional+trauma%2A%E2%80%9D+%5Btiab%5D+OR+%E2%80%9Cchildhood+abus%2A%E2%80%9D%5Btiab%5D+OR+%28ACEs+%5Btiab%5D+AND+child+%5Btiab%5D%29+OR+%28ACE%5Btiab%5D+AND+child%5Btiab%5D%29+OR+%E2%80%9Cchild+physical+abus%2A%E2%80%9D%5Btiab%5D+OR+%E2%80%9Cchildhood+physical+abus%2A%E2%80%9D%5Btiab%5D+OR+%E2%80%9Cearly+life+adversit%2A%E2%80%9D%5Btiab%5D+OR+%E2%80%9CIncest%2A%E2%80%9D%5Btiab%5D&sort=relevance) |
| #9 | Search: **"Stress Disorders, Traumatic"[Mesh] OR "Psychological Distress"[Mesh] OR "complex trauma*"[tiab] OR "posttraumatic stress*"[tiab] OR "post-traumatic stress*"[tiab] OR "posttraumatic-disorder*"[tiab] OR "post-traumatic disorder*"[tiab] OR "post traumatic neuros*"[tiab] OR "posttraumatic neuros*"[tiab] OR "traumatic distress*"[tiab] OR "posttraumatic psychos*"[tiab] OR "post-traumatic psychos*"[tiab] OR "posttraumatic syndrom*"[tiab] OR "post-traumatic syndrom*"[tiab] OR "trauma-and-stressor-related-disorder*"[tiab] OR "traumatic stress disorder*"[tiab] OR ptsd*[tiab] OR ptss*[tiab] OR "combat-disorder*"[tiab] OR "combat-fatigue*"[tiab] OR "combat-stress*"[tiab] OR "shell-shock*"[tiab] OR "combat-neuros*"[tiab] OR "war-neuros*"[tiab] OR "battle-fatigue*"[tiab] OR "psychological stress*"[tiab] OR "psychological trauma*"[tiab] OR psychotrauma*[tiab] OR "psycho-trauma*"[tiab] OR "trauma-focused*"[tiab] OR "psychological distress*"[tiab] OR "emotional trauma*"[tiab] OR "emotional distress*"[tiab] OR "emotional damag*"[tiab] OR "emotional injur*"[tiab] OR "mental damag*"[tiab] OR "mental harm*"[tiab] OR "mental injur*"[tiab] OR "mental trauma*"[tiab] OR "sexual trauma*"[tiab] OR "psychological damag*"[tiab] OR "psychological harm*"[tiab] OR "psychological injur*"[tiab] OR "Posttraumatische Belastungsstörung"[tiab] OR PTBS[tiab]** | [123,010](https://pubmed.ncbi.nlm.nih.gov/?term=longquery0f9a27c35619d5b72043&sort=relevance) |
| #8 | Search: **boulimi*[tiab]** | [37](https://pubmed.ncbi.nlm.nih.gov/?term=boulimi%2A%5Btiab%5D&sort=relevance) |
| #7 | Search: **"Feeding and Eating Disorders"[Mesh] OR "eating disorder*"[tiab] OR "feeding disorder*"[tiab] OR "binge eating*"[tiab] OR "bulimic*"[tiab] OR "bulimia"[tiab] OR "boulimic*"[tiab] OR "anorexi*"[tiab] OR "anorectic"[tiab] OR "underweight*"[tiab] OR "Avoidant Restrictive Food Intake Disorder*"[tiab] OR "Diabulimi*"[tiab] OR "diaboulimi*"[tiab] OR "Food Addiction*"[tiab] OR "Night Eating Syndrome*"[tiab] OR "Orthorexi*"[tiab] OR "Relative Energy Deficienc*"[tiab] OR "Female Athlete Triad Syndrom*" OR "self induced vomit*"[tiab] OR "purging behavio*"[tiab] OR "purging disorder*"[tiab] OR OSFED[tiab] OR EDNOS[tiab] OR ARFID[tiab]** | [90,065](https://pubmed.ncbi.nlm.nih.gov/?term=%22Feeding+and+Eating+Disorders%22%5BMesh%5D+OR+%E2%80%9Ceating+disorder%2A%E2%80%9D%5Btiab%5D+OR+%E2%80%9Cfeeding+disorder%2A%E2%80%9D%5Btiab%5D+OR+%E2%80%9Cbinge+eating%2A%E2%80%9D%5Btiab%5D+OR+%E2%80%9Cbulimic%2A%E2%80%9D%5Btiab%5D+OR+%E2%80%9Cbulimia%E2%80%9D%5Btiab%5D+OR+%E2%80%9Cboulimic%2A%E2%80%9D%5Btiab%5D+OR+%E2%80%9Canorexi%2A%E2%80%9D%5Btiab%5D+OR+%E2%80%9Canorectic%E2%80%9D%5Btiab%5D+OR+%E2%80%9Cunderweight%2A%E2%80%9D%5Btiab%5D+OR+%E2%80%9CAvoidant+Restrictive+Food+Intake+Disorder%2A%E2%80%9D%5Btiab%5D+OR+%E2%80%9CDiabulimi%2A%E2%80%9D%5Btiab%5D+OR+%E2%80%9Cdiaboulimi%2A%E2%80%9D%5Btiab%5D++OR+%E2%80%9CFood+Addiction%2A%E2%80%9D%5Btiab%5D+OR+%E2%80%9CNight+Eating+Syndrome%2A%E2%80%9D%5Btiab%5D+OR+%E2%80%9COrthorexi%2A%E2%80%9D%5Btiab%5D+OR+%E2%80%9CRelative+Energy+Deficienc%2A%E2%80%9D%5Btiab%5D+OR+%E2%80%9CFemale+Athlete+Triad+Syndrom%2A%E2%80%9D+OR+%E2%80%9Cself+induced+vomit%2A%E2%80%9D%5Btiab%5D+OR+%E2%80%9Cpurging+behavio%2A%E2%80%9D%5Btiab%5D+OR+%E2%80%9Cpurging+disorder%2A%E2%80%9D%5Btiab%5D+OR+OSFED%5Btiab%5D+OR+EDNOS%5Btiab%5D+OR+ARFID%5Btiab%5D&sort=relevance) |
| #2 | Search: **"Feeding and Eating Disorders"[Mesh] OR "eating disorder*"[tiab] OR "feeding disorder*"[tiab] OR "binge eating*"[tiab] OR "bulimic*"[tiab] OR "bulimia"[tiab] OR "boulimic*"[tiab] OR "anorexi*"[tiab] OR "anorectic"[tiab] OR "underweight*"[tiab] OR "Avoidant Restrictive Food Intake Disorder*"[tiab] OR "Diabulimi*"[tiab] OR "diaboulimi*"[tiab] OR "Food Addiction*"[tiab] OR "Night Eating Syndrome*"[tiab] OR "Orthorexi*"[tiab] OR "Relative Energy Deficienc*"[tiab] OR "Female Athlete Triad Syndrom*" OR "self induced vomit*"[tiab] OR "purging behavio*"[tiab] OR OSFED[tiab] OR EDNOS[tiab]** | [90,058](https://pubmed.ncbi.nlm.nih.gov/?term=%22Feeding+and+Eating+Disorders%22%5BMesh%5D+OR+%E2%80%9Ceating+disorder%2A%E2%80%9D%5Btiab%5D+OR+%E2%80%9Cfeeding+disorder%2A%E2%80%9D%5Btiab%5D+OR+%E2%80%9Cbinge+eating%2A%E2%80%9D%5Btiab%5D+OR+%E2%80%9Cbulimic%2A%E2%80%9D%5Btiab%5D+OR+%E2%80%9Cbulimia%E2%80%9D%5Btiab%5D+OR+%E2%80%9Cboulimic%2A%E2%80%9D%5Btiab%5D+OR+%E2%80%9Canorexi%2A%E2%80%9D%5Btiab%5D+OR+%E2%80%9Canorectic%E2%80%9D%5Btiab%5D+OR+%E2%80%9Cunderweight%2A%E2%80%9D%5Btiab%5D+OR+%E2%80%9CAvoidant+Restrictive+Food+Intake+Disorder%2A%E2%80%9D%5Btiab%5D+OR+%E2%80%9CDiabulimi%2A%E2%80%9D%5Btiab%5D+OR+%E2%80%9Cdiaboulimi%2A%E2%80%9D%5Btiab%5D++OR+%E2%80%9CFood+Addiction%2A%E2%80%9D%5Btiab%5D+OR+%E2%80%9CNight+Eating+Syndrome%2A%E2%80%9D%5Btiab%5D+OR+%E2%80%9COrthorexi%2A%E2%80%9D%5Btiab%5D+OR+%E2%80%9CRelative+Energy+Deficienc%2A%E2%80%9D%5Btiab%5D+OR+%E2%80%9CFemale+Athlete+Triad+Syndrom%2A%E2%80%9D+OR+%E2%80%9Cself+induced+vomit%2A%E2%80%9D%5Btiab%5D+OR+%E2%80%9Cpurging+behavio%2A%E2%80%9D%5Btiab%5D+OR+OSFED%5Btiab%5D+OR+EDNOS%5Btiab%5D&sort=relevance) |
| #1 | Search: **OSFED[tiab] OR EDNOS[tiab]** Sort by: **Most Recent** | [494](https://pubmed.ncbi.nlm.nih.gov/?sort=date&term=OSFED%5Btiab%5D+OR+EDNOS%5Btiab%5D) |

# Meeting 18-3-2024

| **Search** | **Query** | **Results** |
| --- | --- | --- |
| #7 | Search: **#1 AND #5 AND #6** Sort by: **Most Recent** | [520](https://pubmed.ncbi.nlm.nih.gov/?term=%231+AND+%235+AND+%236&sort=date) |
| #13 | Search: **#1 AND #6** Sort by: **Most Recent** | [25,012](https://pubmed.ncbi.nlm.nih.gov/?term=%231+AND+%236&sort=date) |
| #8 | Search: **oudijn[Author]** Sort by: **Most Recent** | [11](https://pubmed.ncbi.nlm.nih.gov/?sort=date&term=oudijn%5BAuthor%5D&sort_order=desc) |
| #12 | Search: **#8 AND #5** Sort by: **Most Recent** | 0 |
| #11 | Search: **#8 AND #6** Sort by: **Most Recent** | [8](https://pubmed.ncbi.nlm.nih.gov/?term=%238+AND+%236&sort=date) |
| #10 | Search: **#8 AND #1** Sort by: **Most Recent** | [6](https://pubmed.ncbi.nlm.nih.gov/?term=%238+AND+%231&sort=date) |
| #9 | Search: **#8 AND #7** Sort by: **Most Recent** | 0 |
| #6 | Search: **"Antidepressive Agents"[Mesh] OR "Antidepressive Agents" [Pharmacological Action] OR "Anti-Anxiety Agents"[Mesh] OR "Anti-Anxiety Agents" [Pharmacological Action] OR "Tranquilizing Agents"[Mesh] OR "Psychotropic Drugs"[Mesh] OR "Antipsychotic Agents"[Mesh] OR "Antipsychotic Agents" [Pharmacological Action] OR "Drug Therapy"[Mesh] OR "Off-Label Use"[Mesh] OR "Drug Repositioning"[Mesh] OR "Therapies, Investigational"[Mesh] OR "Deep Brain Stimulation"[Mesh] OR "Transcranial Magnetic Stimulation"[Mesh] OR "Transcranial Direct Current Stimulation"[Mesh] OR "Neurofeedback"[Mesh] OR "Hormone Replacement Therapy"[Mesh] OR "Hydrocortisone"[Mesh] OR "Oxytocin"[Mesh] OR "Epinephrine"[Mesh] OR "Norepinephrine"[Mesh] OR "Androgens"[Mesh] OR "Androgens" [Pharmacological Action] OR "Estrogens"[Mesh] OR "Estrogens" [Pharmacological Action] OR "Melatonin"[Mesh] OR "Leptin"[Mesh] OR "Ghrelin"[Mesh] OR "Exercise Movement Techniques"[Mesh] OR "Phenethylamines"[Mesh] OR "Mind-Body Therapies"[Mesh] OR "Complementary Therapies"[Mesh] OR "Acupuncture Therapy"[Mesh] OR "Phytotherapy"[Mesh] OR "Homeopathy"[Mesh] OR "Medicine, Traditional"[Mesh] OR "Holistic Health"[Mesh] OR "General Surgery"[Mesh] OR "Surgical Procedures, Operative"[Mesh] OR "Bariatric Surgery"[Mesh] OR "Electroconvulsive Therapy"[Mesh] OR "Magnetic Field Therapy"[Mesh] OR "Hyperbaric Oxygenation"[Mesh] OR "Life Style"[Mesh] OR "Dietary Supplements"[Mesh] OR "dietary supplement, SPORT" [Supplementary Concept] OR "Nutritional Support"[Mesh] OR Medication*[tiab] OR Antidepressant*[tiab] OR "anti-depressant*"[tiab] OR "Anti-anxiety agent*"[tiab] OR Antipsychotic*[tiab] OR "Mood stabilizer*"[tiab] OR tranquilizer*[tiab] OR Pharmacotherap*[tiab] OR "Pharmacological Intervention*"[tiab] OR "Psychotropic drug*"[tiab] OR "Off-label*"[tiab] OR repurposing[tiab] OR "Experimental drug*"[tiab] OR "Biological Intervention*"[tiab] OR "Deep brain stimulation"[tiab] OR "Transcranial magnetic stimulation"[tiab] OR TMS[tiab] OR "Transcranial direct current stimulation"[tiab] OR tDCS[tiab] OR Neurofeedback[tiab] OR "Endocrine Intervention*"[tiab] OR "Hormone therap*"[tiab] OR "Thyroid intervention*"[tiab] OR "Endocrine modulator*"[tiab] OR "Oxytocin"[tiab] OR "Epinephrine"[tiab] OR "Norepinephrine"[tiab] OR Insulin[tiab] OR Cortisol[tiab] OR Androgen[tiab] OR Estrogen[tiab] OR Melatonin[tiab] OR Leptin[tiab] OR Ghrelin[tiab] OR "Lifestyle intervention*"[tiab] OR "Exercise therap*"[tiab] OR Yoga[tiab] OR "Tai chi"[tiab] OR "Tai Ji"[tiab] OR "Dance therap*"[tiab] OR "Substance-assisted therap*"[tiab] OR "MDMA-assisted therap*"[tiab] OR "Phenethylamin*"[tiab] OR "Amphetamin*"[tiab] OR "Alternative Complementary Therapies"[tiab:~3] OR Acupuncture[tiab] OR "Herbal remedie*"[tiab] OR Homeopath*[tiab] OR "traditional medicine"[tiab] OR "Holistic health intervention*"[tiab] OR "Medical Intervention*"[tiab] OR Surgery[tiab] OR "Electroconvulsive therap*"[tiab] OR ECT[tiab] OR "Electromagnetic therap*"[tiab] OR "Repetitive transcranial magnetic stimulation*"[tiab] OR rTMS[tiab] OR "Hypobaric oxygen therapy"[tiab:~3] OR "Sleep intervention*"[tiab] OR "Stress management"[tiab] OR "Dietary chang*"[tiab] OR "Nutritional intervention*"[tiab] OR "Dietary supplement*"[tiab] OR "Nutritional counsel*"[tiab] OR "Dietary intervention*"[tiab] OR "Nutrient supplementation*"[tiab] OR "Nutraceutical intervention*"[tiab]** Sort by: **Most Recent** | [7,710,625](https://pubmed.ncbi.nlm.nih.gov/?sort=date&term=%22Antidepressive+Agents%22%5BMesh%5D+OR+%22Antidepressive+Agents%22+%5BPharmacological+Action%5D+OR+%22Anti-Anxiety+Agents%22%5BMesh%5D+OR+%22Anti-Anxiety+Agents%22+%5BPharmacological+Action%5D+OR+%22Tranquilizing+Agents%22%5BMesh%5D+OR+%22Psychotropic+Drugs%22%5BMesh%5D+OR+%22Antipsychotic+Agents%22%5BMesh%5D+OR+%22Antipsychotic+Agents%22+%5BPharmacological+Action%5D+OR+%22Drug+Therapy%22%5BMesh%5D+OR+%22Off-Label+Use%22%5BMesh%5D+OR+%22Drug+Repositioning%22%5BMesh%5D+OR+%22Therapies%2C+Investigational%22%5BMesh%5D+OR+%22Deep+Brain+Stimulation%22%5BMesh%5D+OR+%22Transcranial+Magnetic+Stimulation%22%5BMesh%5D+OR+%22Transcranial+Direct+Current+Stimulation%22%5BMesh%5D+OR+%22Neurofeedback%22%5BMesh%5D+OR+%22Hormone+Replacement+Therapy%22%5BMesh%5D+OR+%22Hydrocortisone%22%5BMesh%5D+OR+%22Oxytocin%22%5BMesh%5D+OR+%22Epinephrine%22%5BMesh%5D+OR+%22Norepinephrine%22%5BMesh%5D+OR+%22Androgens%22%5BMesh%5D+OR+%22Androgens%22+%5BPharmacological+Action%5D+OR+%22Estrogens%22%5BMesh%5D+OR+%22Estrogens%22+%5BPharmacological+Action%5D+OR+%22Melatonin%22%5BMesh%5D+OR+%22Leptin%22%5BMesh%5D+OR+%22Ghrelin%22%5BMesh%5D+OR+%22Exercise+Movement+Techniques%22%5BMesh%5D+OR+%22Phenethylamines%22%5BMesh%5D+OR+%22Mind-Body+Therapies%22%5BMesh%5D+OR+%22Complementary+Therapies%22%5BMesh%5D+OR+%22Acupuncture+Therapy%22%5BMesh%5D+OR+%22Phytotherapy%22%5BMesh%5D+OR+%22Homeopathy%22%5BMesh%5D+OR+%22Medicine%2C+Traditional%22%5BMesh%5D+OR+%22Holistic+Health%22%5BMesh%5D+OR+%22General+Surgery%22%5BMesh%5D+OR+%22Surgical+Procedures%2C+Operative%22%5BMesh%5D+OR+%22Bariatric+Surgery%22%5BMesh%5D+OR+%22Electroconvulsive+Therapy%22%5BMesh%5D+OR+%22Magnetic+Field+Therapy%22%5BMesh%5D+OR+%22Hyperbaric+Oxygenation%22%5BMesh%5D+OR+%22Life+Style%22%5BMesh%5D+OR+%22Dietary+Supplements%22%5BMesh%5D+OR++%22dietary+supplement%2C+SPORT%22+%5BSupplementary+Concept%5D+OR+%22Nutritional+Support%22%5BMesh%5D+OR+Medication%2A%5Btiab%5D+OR+Antidepressant%2A%5Btiab%5D+OR+%E2%80%9Canti-depressant%2A%E2%80%9D%5Btiab%5D+OR+%E2%80%9CAnti-anxiety+agent%2A%E2%25) |
| #5 | Search: **#2 OR #4** Sort by: **Most Recent** | [262,451](https://pubmed.ncbi.nlm.nih.gov/?term=%232+OR+%234&sort=date) |
| #4 | Search: **"Adult Survivors of Child Adverse Events"[Mesh] OR "Battered Child Syndrome"[Mesh] OR "Child Abuse"[Mesh] OR "Adverse Childhood Experiences"[Mesh] OR "Emotional Abuse"[Mesh] OR "Incest"[Mesh] OR "Sex Offenses"[Mesh] OR "Intimate Partner Violence"[Mesh] OR "Violence"[Mesh] OR "childhood trauma*"[tiab] OR "childhood adverse event*"[tiab] OR "childhood adversit*"[tiab] OR "adverse childhood experien*"[tiab] OR "battered child*"[tiab] OR "child abus*"[tiab] OR "child sexual abus*"[tiab] OR "childhood sexual abus*"[tiab] OR "child maltreatment*"[tiab] OR "childhood maltreatment*"[tiab] OR "child mistreatment*"[tiab] OR "child neglect*"[tiab] OR "childhood neglect*"[tiab] OR "childhood psychological trauma*"[tiab] OR "childhood emotional trauma*" [tiab] OR "childhood abus*"[tiab] OR (ACEs [tiab] AND child [tiab]) OR (ACE[tiab] AND child[tiab]) OR "child physical abus*"[tiab] OR "childhood physical abus*"[tiab] OR "early life adversit*"[tiab] OR "Incest*"[tiab] OR "sex offense*"[tiab] OR "sexual abus*"[tiab] OR "physical abus*"[tiab] OR "intimate partner violen*"[tiab] OR "spouse abus*"[tiab] OR rape*[tiab]** Sort by: **Most Recent** | [154,253](https://pubmed.ncbi.nlm.nih.gov/?sort=date&term=%22Adult+Survivors+of+Child+Adverse+Events%22%5BMesh%5D+OR+%22Battered+Child+Syndrome%22%5BMesh%5D+OR+%22Child+Abuse%22%5BMesh%5D+OR+%22Adverse+Childhood+Experiences%22%5BMesh%5D+OR+%22Emotional+Abuse%22%5BMesh%5D+OR+%E2%80%9CIncest%E2%80%9D%5BMesh%5D+OR+%22Sex+Offenses%22%5BMesh%5D+OR+%22Intimate+Partner+Violence%22%5BMesh%5D+OR+%22Violence%22%5BMesh%5D+OR+%E2%80%9Cchildhood+trauma%2A%E2%80%9D%5Btiab%5D+OR+%E2%80%9Cchildhood+adverse+event%2A%E2%80%9D%5Btiab%5D+OR+%E2%80%9Cchildhood+adversit%2A%E2%80%9D%5Btiab%5D+OR+%E2%80%9Cadverse+childhood+experien%2A%E2%80%9D%5Btiab%5D+OR+%E2%80%9Cbattered+child%2A%E2%80%9D%5Btiab%5D+OR+%E2%80%9Cchild+abus%2A%E2%80%9D%5Btiab%5D+OR+%E2%80%9Cchild+sexual+abus%2A%E2%80%9D%5Btiab%5D+OR+%E2%80%9Cchildhood+sexual+abus%2A%E2%80%9D%5Btiab%5D+OR+%E2%80%9Cchild+maltreatment%2A%E2%80%9D%5Btiab%5D+OR+%E2%80%9Cchildhood+maltreatment%2A%E2%80%9D%5Btiab%5D+OR+%E2%80%9Cchild+mistreatment%2A%E2%80%9D%5Btiab%5D+OR+%E2%80%9Cchild+neglect%2A%E2%80%9D%5Btiab%5D+OR+%E2%80%9Cchildhood+neglect%2A%E2%80%9D%5Btiab%5D+OR+%E2%80%9Cchildhood+psychological+trauma%2A%E2%80%9D%5Btiab%5D+OR+%E2%80%9Cchildhood+emotional+trauma%2A%E2%80%9D+%5Btiab%5D+OR+%E2%80%9Cchildhood+abus%2A%E2%80%9D%5Btiab%5D+OR+%28ACEs+%5Btiab%5D+AND+child+%5Btiab%5D%29+OR+%28ACE%5Btiab%5D+AND+child%5Btiab%5D%29+OR+%E2%80%9Cchild+physical+abus%2A%E2%80%9D%5Btiab%5D+OR+%E2%80%9Cchildhood+physical+abus%2A%E2%80%9D%5Btiab%5D+OR+%E2%80%9Cearly+life+adversit%2A%E2%80%9D%5Btiab%5D+OR+%E2%80%9CIncest%2A%E2%80%9D%5Btiab%5D+OR+%E2%80%9Csex+offense%2A%E2%80%9D%5Btiab%5D+OR+%E2%80%9Csexual+abus%2A%E2%80%9D%5Btiab%5D+OR+%E2%80%9Cphysical+abus%2A%E2%80%9D%5Btiab%5D+OR+%E2%80%9Cintimate+partner+violen%2A%E2%80%9D%5Btiab%5D+OR+%E2%80%9Cspouse+abus%2A%E2%80%9D%5Btiab%5D+OR+rape%2A%5Btiab%5D) |
| #2 | Search: **"Stress Disorders, Traumatic"[Mesh] OR "Psychological Distress"[Mesh] OR "complex trauma*"[tiab] OR "posttraumatic stress*"[tiab] OR "post-traumatic stress*"[tiab] OR "posttraumatic-disorder*"[tiab] OR "post-traumatic disorder*"[tiab] OR "post traumatic neuros*"[tiab] OR "posttraumatic neuros*"[tiab] OR "traumatic distress*"[tiab] OR "posttraumatic psychos*"[tiab] OR "post-traumatic psychos*"[tiab] OR "posttraumatic syndrom*"[tiab] OR "post-traumatic syndrom*"[tiab] OR "trauma-and-stressor-related-disorder*"[tiab] OR "traumatic stress disorder*"[tiab] OR ptsd*[tiab] OR ptss*[tiab] OR "combat-disorder*"[tiab] OR "combat-fatigue*"[tiab] OR "combat-stress*"[tiab] OR "shell-shock*"[tiab] OR "combat-neuros*"[tiab] OR "war-neuros*"[tiab] OR "battle-fatigue*"[tiab] OR "psychological stress*"[tiab] OR "psychological trauma*"[tiab] OR psychotrauma*[tiab] OR "psycho-trauma*"[tiab] OR "trauma-focused*"[tiab] OR "psychological distress*"[tiab] OR "emotional trauma*"[tiab] OR "emotional distress*"[tiab] OR "emotional damag*"[tiab] OR "emotional injur*"[tiab] OR "mental damag*"[tiab] OR "mental harm*"[tiab] OR "mental injur*"[tiab] OR "mental trauma*"[tiab] OR "sexual trauma*"[tiab] OR "psychological damag*"[tiab] OR "psychological harm*"[tiab] OR "psychological injur*"[tiab] OR "Posttraumatische Belastungsstörung"[tiab] OR PTBS[tiab]** Sort by: **Most Recent** | [123,401](https://pubmed.ncbi.nlm.nih.gov/?term=longquery0f9a27c35619d5b72043&sort=date) |
| #1 | Search: **"Feeding and Eating Disorders"[Mesh] OR "eating disorder*"[tiab] OR "feeding disorder*"[tiab] OR "binge eating*"[tiab] OR "bulimic*"[tiab] OR "bulimia"[tiab] OR "boulimic*"[tiab] OR "anorexi*"[tiab] OR "anorectic"[tiab] OR "underweight*"[tiab] OR "Avoidant Restrictive Food Intake Disorder*"[tiab] OR "Diabulimi*"[tiab] OR "diaboulimi*"[tiab] OR "Food Addiction*"[tiab] OR "Night Eating Syndrome*"[tiab] OR "Orthorexi*"[tiab] OR "Relative Energy Deficienc*"[tiab] OR "Female Athlete Triad Syndrom*" OR "self induced vomit*"[tiab] OR "purging behavio*"[tiab] OR "purging disorder*"[tiab] OR OSFED[tiab] OR EDNOS[tiab] OR ARFID[tiab]** Sort by: **Most Recent** | [90,310](https://pubmed.ncbi.nlm.nih.gov/?sort=date&term=%22Feeding+and+Eating+Disorders%22%5BMesh%5D+OR+%E2%80%9Ceating+disorder%2A%E2%80%9D%5Btiab%5D+OR+%E2%80%9Cfeeding+disorder%2A%E2%80%9D%5Btiab%5D+OR+%E2%80%9Cbinge+eating%2A%E2%80%9D%5Btiab%5D+OR+%E2%80%9Cbulimic%2A%E2%80%9D%5Btiab%5D+OR+%E2%80%9Cbulimia%E2%80%9D%5Btiab%5D+OR+%E2%80%9Cboulimic%2A%E2%80%9D%5Btiab%5D+OR+%E2%80%9Canorexi%2A%E2%80%9D%5Btiab%5D+OR+%E2%80%9Canorectic%E2%80%9D%5Btiab%5D+OR+%E2%80%9Cunderweight%2A%E2%80%9D%5Btiab%5D+OR+%E2%80%9CAvoidant+Restrictive+Food+Intake+Disorder%2A%E2%80%9D%5Btiab%5D+OR+%E2%80%9CDiabulimi%2A%E2%80%9D%5Btiab%5D+OR+%E2%80%9Cdiaboulimi%2A%E2%80%9D%5Btiab%5D+%0AOR+%E2%80%9CFood+Addiction%2A%E2%80%9D%5Btiab%5D+OR+%E2%80%9CNight+Eating+Syndrome%2A%E2%80%9D%5Btiab%5D+OR+%E2%80%9COrthorexi%2A%E2%80%9D%5Btiab%5D+OR+%E2%80%9CRelative+Energy+Deficienc%2A%E2%80%9D%5Btiab%5D+OR+%E2%80%9CFemale+Athlete+Triad+Syndrom%2A%E2%80%9D+OR+%E2%80%9Cself+induced+vomit%2A%E2%80%9D%5Btiab%5D+OR+%E2%80%9Cpurging+behavio%2A%E2%80%9D%5Btiab%5D+OR+%E2%80%9Cpurging+disorder%2A%E2%80%9D%5Btiab%5D+OR+OSFED%5Btiab%5D+OR+EDNOS%5Btiab%5D+OR+ARFID%5Btiab%5D) |

# Definitieve search … (datum)

## PubMed

### Zoekblokken:

**# Anorexia nervosa**

"Anorexia"[Mesh] OR "Anorexia Nervosa"[Mesh] OR “anorexi*”[tiab] OR “anorectic”[tiab] OR “underweight*”[tiab]

**# PTSD – trauma**

"Stress Disorders, Traumatic"[Mesh] OR "Psychological Distress"[Mesh] OR “complex trauma*”[tiab] OR “posttraumatic stress*”[tiab] OR “post-traumatic stress*”[tiab] OR “posttraumatic-disorder*”[tiab] OR “post-traumatic disorder*”[tiab] OR "post traumatic neuros*"[tiab] OR "posttraumatic neuros*"[tiab] OR “traumatic distress*”[tiab] OR "posttraumatic psychos*"[tiab] OR "post-traumatic psychos*"[tiab] OR "posttraumatic syndrom*"[tiab] OR "post-traumatic syndrom*"[tiab] OR "trauma-and-stressor-related-disorder*"[tiab] OR “traumatic stress disorder*”[tiab] OR ptsd*[tiab] OR ptss*[tiab] OR “combat-disorder*”[tiab] OR “combat-fatigue*”[tiab] OR “combat-stress*”[tiab] OR “shell-shock*”[tiab] OR “combat-neuros*”[tiab] OR “war-neuros*”[tiab] OR “battle-fatigue*”[tiab] OR “psychological stress*”[tiab] OR “psychological trauma*”[tiab] OR psychotrauma*[tiab] OR “psycho-trauma*”[tiab] OR “trauma-focused*”[tiab] OR “psychological distress*”[tiab] OR “emotional trauma*”[tiab] OR “emotional distress*”[tiab] OR “emotional damag*”[tiab] OR “emotional injur*”[tiab] OR “mental damag*”[tiab] OR “mental harm*”[tiab] OR “mental injur*”[tiab] OR “mental trauma*”[tiab] OR “sexual trauma*”[tiab] OR “psychological damag*”[tiab] OR “psychological harm*”[tiab] OR “psychological injur*”[tiab] OR “Posttraumatische Belastungsstörung”[tiab] OR PTBS[tiab]

**# Treatment (non-psychological)**

### Results 286 records

| Search | Query | Results |
| --- | --- | --- |
| #4 | Search: #1 AND #2 AND #3 Sort by: Most Recent | [286](https://pubmed.ncbi.nlm.nih.gov/?term=%231+AND+%232+AND+%234&sort=date) |
| #3 |  |  |
| #2 | Search: "Stress Disorders, Traumatic"[Mesh] OR "Psychological Distress"[Mesh] OR "complex trauma*"[tiab] OR "posttraumatic stress*"[tiab] OR "post-traumatic stress*"[tiab] OR "posttraumatic-disorder*"[tiab] OR "post-traumatic disorder*"[tiab] OR "post traumatic neuros*"[tiab] OR "posttraumatic neuros*"[tiab] OR "traumatic distress*"[tiab] OR "posttraumatic psychos*"[tiab] OR "post-traumatic psychos*"[tiab] OR "posttraumatic syndrom*"[tiab] OR "post-traumatic syndrom*"[tiab] OR "trauma-and-stressor-related-disorder*"[tiab] OR "traumatic stress disorder*"[tiab] OR ptsd*[tiab] OR ptss*[tiab] OR "combat-disorder*"[tiab] OR "combat-fatigue*"[tiab] OR "combat-stress*"[tiab] OR "shell-shock*"[tiab] OR "combat-neuros*"[tiab] OR "war-neuros*"[tiab] OR "battle-fatigue*"[tiab] OR "psychological stress*"[tiab] OR "psychological trauma*"[tiab] OR psychotrauma*[tiab] OR "psycho-trauma*"[tiab] OR "trauma-focused*"[tiab] OR "psychological distress*"[tiab] OR "emotional trauma*"[tiab] OR "emotional distress*"[tiab] OR "emotional damag*"[tiab] OR "emotional injur*"[tiab] OR "mental damag*"[tiab] OR "mental harm*"[tiab] OR "mental injur*"[tiab] OR "mental trauma*"[tiab] OR "sexual trauma*"[tiab] OR "psychological damag*"[tiab] OR "psychological harm*"[tiab] OR "psychological injur*"[tiab] OR "Posttraumatische Belastungsstörung"[tiab] OR PTBS[tiab] Sort by: Most Recent | [116,833](https://pubmed.ncbi.nlm.nih.gov/?term=longquery0f9a27c35619d5b72043&sort=date) |
| #1 | Search: "Anorexia"[Mesh] OR "Anorexia Nervosa"[Mesh] OR "anorexi*"[tiab] OR "anorectic"[tiab] OR "underweight*"[tiab] Sort by: Most Recent | [58,821](https://pubmed.ncbi.nlm.nih.gov/?term=%22Anorexia%22%5BMesh%5D+OR+%22Anorexia+Nervosa%22%5BMesh%5D+OR+%E2%80%9Canorexi%2A%E2%80%9D%5Btiab%5D+OR+%E2%80%9Canorectic%E2%80%9D%5Btiab%5D+OR+%E2%80%9Cunderweight%2A%E2%80%9D%5Btiab%5D+&sort=date) |

## Embase (Embase.com)

Including conference abstracts and papers Search Blocks

**# Anorexia nervosa**

'anorexia'/exp OR 'anorexia nervosa'/exp OR ‘anorexi*’:ab,ti,kw OR ‘anorectic’:ab,ti,kw OR ‘underweight*’:ab,ti,kw

**# PTSD – trauma**

'posttraumatic stress disorder'/exp OR 'distress syndrome'/exp OR ‘complex trauma*’:ab,ti,kw OR ‘posttraumatic stress*’:ab,ti,kw OR ‘post-traumatic stress*’:ab,ti,kw OR ‘posttraumatic-disorder*’:ab,ti,kw OR ‘post-traumatic disorder*’:ab,ti,kw OR ‘post traumatic neuros*’:ab,ti,kw OR ‘posttraumatic neuros*’:ab,ti,kw OR ‘traumatic distress*’:ab,ti,kw OR ‘posttraumatic psychos*’:ab,ti,kw OR ‘post-traumatic psychos*’:ab,ti,kw OR ‘posttraumatic syndrom*’:ab,ti,kw OR ‘post-traumatic syndrom*’:ab,ti,kw OR ‘trauma-and-stressor-related-disorder*’:ab,ti,kw OR ‘traumatic stress disorder*’:ab,ti,kw OR ptsd*:ab,ti,kw OR ptss*:ab,ti,kw OR ‘combat-disorder*’:ab,ti,kw OR ‘combat-fatigue*’:ab,ti,kw OR ‘combat-stress*’:ab,ti,kw OR ‘shell-shock*’:ab,ti,kw OR ‘combat-neuros*’:ab,ti,kw OR ‘war-neuros*’:ab,ti,kw OR ‘battle-fatigue*’:ab,ti,kw OR ‘psychological stress*’:ab,ti,kw OR ‘psychological trauma*’:ab,ti,kw OR psychotrauma*:ab,ti,kw OR ‘psycho-trauma*’:ab,ti,kw OR ‘trauma-focused*’:ab,ti,kw OR ‘psychological distress*’:ab,ti,kw OR ‘emotional trauma*’:ab,ti,kw OR ‘emotional distress*’:ab,ti,kw OR ‘emotional damag*’:ab,ti,kw OR ‘emotional injur*’:ab,ti,kw OR ‘mental damag*’:ab,ti,kw OR ‘mental harm*’:ab,ti,kw OR ‘mental injur*’:ab,ti,kw OR ‘mental trauma*’:ab,ti,kw OR ‘sexual trauma*’:ab,ti,kw OR ‘psychological damag*’:ab,ti,kw OR ‘psychological harm*’:ab,ti,kw OR ‘psychological injur*’:ab,ti,kw OR ‘Posttraumatische Belastungsstörung’:ab,ti,kw OR PTBS:ab,ti,kw

**# Treatment**

### Results 1.256 records

Anorexia AND PTSD AND therapy 🡪 1.256

| **No.** | **Query** | **Results** |
| --- | --- | --- |
| **#4** | **#1** AND **#2** AND **#3** | **1,256** |
| **#3** |  |  |
| **#2** | **'posttraumatic stress disorder'**/exp OR **'distress syndrome'**/exp OR **'complex trauma*'**:ab,ti,kw OR **'posttraumatic stress*'**:ab,ti,kw OR **'post-traumatic stress*'**:ab,ti,kw OR **'posttraumatic-disorder*'**:ab,ti,kw OR **'post-traumatic disorder*'**:ab,ti,kw OR **'post traumatic neuros*'**:ab,ti,kw OR **'posttraumatic neuros*'**:ab,ti,kw OR **'traumatic distress*'**:ab,ti,kw OR **'posttraumatic psychos*'**:ab,ti,kw OR **'post-traumatic psychos*'**:ab,ti,kw OR **'posttraumatic syndrom*'**:ab,ti,kw OR **'post-traumatic syndrom*'**:ab,ti,kw OR **'trauma-and-stressor-related-disorder*'**:ab,ti,kw OR **'traumatic stress disorder*'**:ab,ti,kw OR **ptsd***:ab,ti,kw OR **ptss***:ab,ti,kw OR **'combat-disorder*'**:ab,ti,kw OR **'combat-fatigue*'**:ab,ti,kw OR **'combat-stress*'**:ab,ti,kw OR **'shell-shock*'**:ab,ti,kw OR **'combat-neuros*'**:ab,ti,kw OR **'war-neuros*'**:ab,ti,kw OR **'battle-fatigue*'**:ab,ti,kw OR **'psychological stress*'**:ab,ti,kw OR **'psychological trauma*'**:ab,ti,kw OR **psychotrauma***:ab,ti,kw OR **'psycho-trauma*'**:ab,ti,kw OR **'trauma-focused*'**:ab,ti,kw OR **'psychological distress*'**:ab,ti,kw OR **'emotional trauma*'**:ab,ti,kw OR **'emotional distress*'**:ab,ti,kw OR **'emotional damag*'**:ab,ti,kw OR **'emotional injur*'**:ab,ti,kw OR **'mental damag*'**:ab,ti,kw OR **'mental harm*'**:ab,ti,kw OR **'mental injur*'**:ab,ti,kw OR **'mental trauma*'**:ab,ti,kw OR **'sexual trauma*'**:ab,ti,kw OR **'psychological damag*'**:ab,ti,kw OR **'psychological harm*'**:ab,ti,kw OR **'psychological injur*'**:ab,ti,kw OR **'posttraumatische belastungsstörung'**:ab,ti,kw OR **ptbs**:ab,ti,kw | **193,997** |
| **#1** | **'anorexia'**/exp OR **'anorexia nervosa'**/exp OR **'anorexi*'**:ab,ti,kw OR **'anorectic'**:ab,ti,kw OR **'underweight*'**:ab,ti,kw | **133,733** |

## APA PsycInfo (EBSCO)

Proximity operator W1

### Zoekblokken

**# Anorexia nervosa**

DE "Underweight" OR DE "Anorexia Nervosa" OR TI(“anorexi*” OR “anorectic” OR “underweight*”) OR AB(“anorexi*” OR “anorectic” OR “underweight*”) OR KW(“anorexi*” OR “anorectic” OR “underweight*”)

**# PTSD – trauma**

DE "Complex Trauma" OR DE "Emotional Trauma" OR DE "Posttraumatic Stress" OR DE "Complex PTSD" OR DE "DESNOS" OR DE "Posttraumatic Stress Disorder" OR DE "Stress and Trauma Related Disorders" OR DE "Psychological Stress" OR TI(“complex trauma*” OR “posttraumatic stress*” OR “post-traumatic stress*” OR “posttraumatic-disorder*” OR “post-traumatic disorder*” OR "post traumatic neuros*" OR "posttraumatic neuros*" OR “traumatic distress*” OR "posttraumatic psychos*" OR "post-traumatic psychos*" OR "posttraumatic syndrom*" OR "post-traumatic syndrom*" OR "trauma-and-stressor-related-disorder*" OR “traumatic stress disorder*” OR ptsd* OR ptss* OR “combat-disorder*” OR “combat-fatigue*” OR “combat-stress*” OR “shell-shock*” OR “combat-neuros*” OR “war-neuros*” OR “battle-fatigue*” OR “psychological stress*” OR “psychological trauma*” OR psychotrauma* OR “psycho-trauma*” OR “trauma-focused*” OR “psychological distress*” OR “emotional trauma*” OR “emotional distress*” OR “emotional damag*” OR “emotional injur*” OR “mental damag*” OR “mental harm*” OR “mental injur*” OR “mental trauma*” OR “sexual trauma*” OR “psychological damag*” OR “psychological harm*” OR “psychological injur*” OR “Posttraumatische Belastungsstörung” OR PTBS) OR AB(“complex trauma*” OR “posttraumatic stress*” OR “post-traumatic stress*” OR “posttraumatic-disorder*” OR “post-traumatic disorder*” OR "post traumatic neuros*" OR "posttraumatic neuros*" OR “traumatic distress*” OR "posttraumatic psychos*" OR "post-traumatic psychos*" OR "posttraumatic syndrom*" OR "post-traumatic syndrom*" OR "trauma-and-stressor-related-disorder*" OR “traumatic stress disorder*” OR ptsd* OR ptss* OR “combat-disorder*” OR “combat-fatigue*” OR “combat-stress*” OR “shell-shock*” OR “combat-neuros*” OR “war-neuros*” OR “battle-fatigue*” OR “psychological stress*” OR “psychological trauma*” OR psychotrauma* OR “psycho-trauma*” OR “trauma-focused*” OR “psychological distress*” OR “emotional trauma*” OR “emotional distress*” OR “emotional damag*” OR “emotional injur*” OR “mental damag*” OR “mental harm*” OR “mental injur*” OR “mental trauma*” OR “sexual trauma*” OR “psychological damag*” OR “psychological harm*” OR “psychological injur*” OR “Posttraumatische Belastungsstörung” OR PTBS) OR KW(“complex trauma*” OR “posttraumatic stress*” OR “post-traumatic stress*” OR “posttraumatic-disorder*” OR “post-traumatic disorder*” OR "post traumatic neuros*" OR "posttraumatic neuros*" OR “traumatic distress*” OR "posttraumatic psychos*" OR "post-traumatic psychos*" OR "posttraumatic syndrom*" OR "post-traumatic syndrom*" OR "trauma-and-stressor-related-disorder*" OR “traumatic stress disorder*” OR ptsd* OR ptss* OR “combat-disorder*” OR “combat-fatigue*” OR “combat-stress*” OR “shell-shock*” OR “combat-neuros*” OR “war-neuros*” OR “battle-fatigue*” OR “psychological stress*” OR “psychological trauma*” OR psychotrauma* OR “psycho-trauma*” OR “trauma-focused*” OR “psychological distress*” OR “emotional trauma*” OR “emotional distress*” OR “emotional damag*” OR “emotional injur*” OR “mental damag*” OR “mental harm*” OR “mental injur*” OR “mental trauma*” OR “sexual trauma*” OR “psychological damag*” OR “psychological harm*” OR “psychological injur*” OR “Posttraumatische Belastungsstörung” OR PTBS)

**# Treatment non-psychological**

### Results 211 records

| **#** | **Query** | **Limiters/Expanders** | **Results** |
| --- | --- | --- | --- |
| S5 | S4 | Limiters –  Academic journals | 211 |
| S4 | S1 AND S2 AND S3 | Search modes - Boolean/Phrase | 260 |
| S3 |  | Search modes - Boolean/Phrase | 1,903,869 |
| S2 | DE "Complex Trauma" OR DE "Emotional Trauma" OR DE "Posttraumatic Stress" OR DE "Complex PTSD" OR DE "DESNOS" OR DE "Posttraumatic Stress Disorder" OR DE "Stress and Trauma Related Disorders" OR DE "Psychological Stress" OR TI(“complex trauma*” OR “posttraumatic stress*” OR “post-traumatic stress*” OR “posttraumatic-disorder*” OR “post-traumatic disorder*” OR "post traumatic neuros*" OR "posttraumatic neuros*" OR “traumatic distress*” OR "posttraumatic psychos*" OR "post-traumatic psychos*" OR "posttraumatic syndrom*" OR "post-traumatic syndrom*" OR "trauma-and-stressor-related-disorder*" OR “traumatic stress disorder*” OR ptsd* OR ptss* OR “combat-disorder*” OR “combat-fatigue*” OR “combat-stress*” OR “shell-shock*” OR “combat-neuros*” OR “war-neuros*” OR “battle-fatigue*” OR “psychological stress*” OR “psychological trauma*” OR psychotrauma* OR “psycho-trauma*” OR “trauma-focused*” OR “psychological distress*” OR “emotional trauma*” OR “emotional distress*” OR “emotional damag*” OR “emotional injur*” OR “mental damag*” OR “mental harm*” OR “mental injur*” OR “mental trauma*” OR “sexual trauma*” OR “psychological damag*” OR “psychological harm*” OR “psychological injur*” OR “Posttraumatische Belastungsstörung” OR PTBS) OR AB(“complex trauma*” OR “posttraumatic stress*” OR “post-traumatic stress*” OR “posttraumatic-disorder*” OR “post-traumatic disorder*” OR "post traumatic neuros*" OR "posttraumatic neuros*" OR “traumatic distress*” OR "posttraumatic psychos*" OR "post-traumatic psychos*" OR "posttraumatic syndrom*" OR "post-traumatic syndrom*" OR "trauma-and-stressor-related-disorder*" OR “traumatic stress disorder*” OR ptsd* OR ptss* OR “combat-disorder*” OR “combat-fatigue*” OR “combat-stress*” OR “shell-shock*” OR “combat-neuros*” OR “war-neuros*” OR “battle-fatigue*” OR “psychological stress*” OR “psychological trauma*” OR psychotrauma* OR “psycho-trauma*” OR “trauma-focused*” OR “psychological distress*” OR “emotional trauma*” OR “emotional distress*” OR “emotional damag*” OR “emotional injur*” OR “mental damag*” OR “mental harm*” OR “mental injur*” OR “mental trauma*” OR “sexual trauma*” OR “psychological damag*” OR “psychological harm*” OR “psychological injur*” OR “Posttraumatische Belastungsstörung” OR PTBS) OR KW(“complex trauma*” OR “posttraumatic stress*” OR “post-traumatic stress*” OR “posttraumatic-disorder*” OR “post-traumatic disorder*” OR "post traumatic neuros*" OR "posttraumatic neuros*" OR “traumatic distress*” OR "posttraumatic psychos*" OR "post-traumatic psychos*" OR "posttraumatic syndrom*" OR "post-traumatic syndrom*" OR "trauma-and-stressor-related-disorder*" OR “traumatic stress disorder*” OR ptsd* OR ptss* OR “combat-disorder*” OR “combat-fatigue*” OR “combat-stress*” OR “shell-shock*” OR “combat-neuros*” OR “war-neuros*” OR “battle-fatigue*” OR “psychological stress*” OR “psychological trauma*” OR psychotrauma* OR “psycho-trauma*” OR “trauma-focused*” OR “psychological distress*” OR “emotional trauma*” OR “emotional distress*” OR “emotional damag*” OR “emotional injur*” OR “mental damag*” OR “mental harm*” OR “mental injur*” OR “mental trauma*” OR “sexual trauma*” OR “psychological damag*” OR “psychological harm*” OR “psychological injur*” OR “Posttraumatische Belastungsstörung” OR PTBS) | Search modes - Boolean/Phrase | 115,165 |
| S1 | DE "Underweight" OR DE "Anorexia Nervosa" OR TI(“anorexi*” OR “anorectic” OR “underweight*”) OR AB(“anorexi*” OR “anorectic” OR “underweight*”) OR KW(“anorexi*” OR “anorectic” OR “underweight*”) | Search modes - Boolean/Phrase | 22,218 |

## Web of Science (Clarivate)

### Search blocks

**# Anorexia nervosa**

TS=(“anorexi*” OR “anorectic” OR “underweight*”)

**# PTSD – trauma**

TS=(“complex trauma*” OR “posttraumatic stress*” OR “post-traumatic stress*” OR “posttraumatic-disorder*” OR “post-traumatic disorder*” OR "post traumatic neuros*" OR "posttraumatic neuros*" OR “traumatic distress*” OR "posttraumatic psychos*" OR "post-traumatic psychos*" OR "posttraumatic syndrom*" OR "post-traumatic syndrom*" OR "trauma-and-stressor-related-disorder*" OR “traumatic stress disorder*” OR ptsd* OR ptss* OR “combat-disorder*” OR “combat-fatigue*” OR “combat-stress*” OR “shell-shock*” OR “combat-neuros*” OR “war-neuros*” OR “battle-fatigue*” OR “psychological stress*” OR “psychological trauma*” OR psychotrauma* OR “psycho-trauma*” OR “trauma-focused*” OR “psychological distress*” OR “emotional trauma*” OR “emotional distress*” OR “emotional damag*” OR “emotional injur*” OR “mental damag*” OR “mental harm*” OR “mental injur*” OR “mental trauma*” OR “sexual trauma*” OR “psychological damag*” OR “psychological harm*” OR “psychological injur*” OR “Posttraumatische Belastungsstörung” OR PTBS)

**# Treatment**

### Results 457 records

| Nr. | Query | Results |
| --- | --- | --- |
| #4 | #1 AND #2 AND #3 | 457 |
| #3 |  |  |
| #2 | TS=(“complex trauma*” OR “posttraumatic stress*” OR “post-traumatic stress*” OR “posttraumatic-disorder*” OR “post-traumatic disorder*” OR "post traumatic neuros*" OR "posttraumatic neuros*" OR “traumatic distress*” OR "posttraumatic psychos*" OR "post-traumatic psychos*" OR "posttraumatic syndrom*" OR "post-traumatic syndrom*" OR "trauma-and-stressor-related-disorder*" OR “traumatic stress disorder*” OR ptsd* OR ptss* OR “combat-disorder*” OR “combat-fatigue*” OR “combat-stress*” OR “shell-shock*” OR “combat-neuros*” OR “war-neuros*” OR “battle-fatigue*” OR “psychological stress*” OR “psychological trauma*” OR psychotrauma* OR “psycho-trauma*” OR “trauma-focused*” OR “psychological distress*” OR “emotional trauma*” OR “emotional distress*” OR “emotional damag*” OR “emotional injur*” OR “mental damag*” OR “mental harm*” OR “mental injur*” OR “mental trauma*” OR “sexual trauma*” OR “psychological damag*” OR “psychological harm*” OR “psychological injur*” OR “Posttraumatische Belastungsstörung” OR PTBS) | 159,288 |
| #1 | TS=(“anorexi*” OR “anorectic” OR “underweight*”) | 65,370 |

## Scopus (Elsevier)

### Search blocks

**# Anorexia nervosa**

TITLE-ABS-KEY(“anorexi*” OR “anorectic” OR “underweight*”)

**# PTSD – trauma**

TITLE-ABS-KEY(“complex trauma*” OR “posttraumatic stress*” OR “post-traumatic stress*” OR “posttraumatic-disorder*” OR “post-traumatic disorder*” OR "post traumatic neuros*" OR "posttraumatic neuros*" OR “traumatic distress*” OR "posttraumatic psychos*" OR "post-traumatic psychos*" OR "posttraumatic syndrom*" OR "post-traumatic syndrom*" OR "trauma-and-stressor-related-disorder*" OR “traumatic stress disorder*” OR ptsd* OR ptss* OR “combat-disorder*” OR “combat-fatigue*” OR “combat-stress*” OR “shell-shock*” OR “combat-neuros*” OR “war-neuros*” OR “battle-fatigue*” OR “psychological stress*” OR “psychological trauma*” OR psychotrauma* OR “psycho-trauma*” OR “trauma-focused*” OR “psychological distress*” OR “emotional trauma*” OR “emotional distress*” OR “emotional damag*” OR “emotional injur*” OR “mental damag*” OR “mental harm*” OR “mental injur*” OR “mental trauma*” OR “sexual trauma*” OR “psychological damag*” OR “psychological harm*” OR “psychological injur*” OR “Posttraumatische Belastungsstörung” OR PTBS)

**# Treatment**

### Results 988 records

Onderkant formulier

| History Count | Search Terms |  |
| --- | --- | --- |
| 4 | #1 AND #2 AND #3 | 988 |
| 3 |  |  |
| 2 | TITLE-ABS-KEY ( "complex trauma*"  OR  "posttraumatic stress*"  OR  "post-traumatic stress*"  OR  "posttraumatic-disorder*"  OR  "post-traumatic disorder*"  OR  "post traumatic neuros*"  OR  "posttraumatic neuros*"  OR  "traumatic distress*"  OR  "posttraumatic psychos*"  OR  "post-traumatic psychos*"  OR  "posttraumatic syndrom*"  OR  "post-traumatic syndrom*"  OR  "trauma-and-stressor-related-disorder*"  OR  "traumatic stress disorder*"  OR  ptsd*  OR  ptss*  OR  "combat-disorder*"  OR  "combat-fatigue*"  OR  "combat-stress*"  OR  "shell-shock*"  OR  "combat-neuros*"  OR  "war-neuros*"  OR  "battle-fatigue*"  OR  "psychological stress*"  OR  "psychological trauma*"  OR  psychotrauma*  OR  "psycho-trauma*"  OR  "trauma-focused*"  OR  "psychological distress*"  OR  "emotional trauma*"  OR  "emotional distress*"  OR  "emotional damag*"  OR  "emotional injur*"  OR  "mental damag*"  OR  "mental harm*"  OR  "mental injur*"  OR  "mental trauma*"  OR  "sexual trauma*"  OR  "psychological damag*"  OR  "psychological harm*"  OR  "psychological injur*"  OR  "Posttraumatische Belastungsstörung"  OR  ptbs ) | 167,693 |
| 1 | TITLE-ABS-KEY ( "anorexi*"  OR  "anorectic"  OR  "underweight*" ) | 134,162 |

## Cochrane CENTRAL (Wiley)

### Search blocks

**# Anorexia nervosa**

(anorexi* OR anorectic OR underweight*):ti,ab,kw

**# PTSD – trauma**

(“complex trauma” “complex traumas” OR “posttraumatic stress” OR “post traumatic stress” OR “posttraumatic disorder” OR “posttraumatic disorders” OR “post traumatic disorder” OR “post traumatic disorders” OR "post traumatic neurose" OR “post traumatic neuroses” OR "posttraumatic neurose" OR “posttraumatic neuroses” OR “traumatic distress” OR "posttraumatic psychose" OR “posttraumatic psychoses” OR "post traumatic psychose" OR “post traumatic psychoses” OR "posttraumatic syndrome" OR “posttraumatic syndrome” OR "post-traumatic syndrome" OR “post-traumatic syndromes” OR "trauma and stressor related disorder" OR “trauma and stressor related disorders” OR “traumatic stress disorder” OR “traumatic stress disorders” OR ptsd* OR ptss* OR “combat disorder” OR “combat disorders” OR “combat fatigue” OR “combat stress” OR “shell shock” OR “combat neurose” OR “combat neuroses” OR “war neurose” OR “war neuroses” OR “battle fatigue” OR “psychological stress” OR “psychological trauma” OR “psychological traumas” OR psychotrauma* OR “psycho-trauma” OR “trauma-focused” OR “psychological distress” OR “emotional trauma” OR “emotional traumas” OR “emotional distress” OR “emotional damage” OR “emotional injury” OR “mental damage” OR “mental harm” OR “mental injury” OR “mental trauma” OR “mental traumas” OR “sexual trauma” OR “psychological damage” OR “psychological harm” OR “psychological injury” OR “Posttraumatische Belastungsstörung” OR PTBS):ti,ab,kw

**# Treatment (non-psychological)**

### Results 55 records

| ID | Search | Hits |
| --- | --- | --- |
| #1 | (anorexi* OR anorectic OR underweight*):ti,ab,kw | 7709 |
| #2 | (“complex trauma” “complex traumas” OR “posttraumatic stress” OR “post traumatic stress” OR “posttraumatic disorder” OR “posttraumatic disorders” OR “post traumatic disorder” OR “post traumatic disorders” OR "post traumatic neurose" OR “post traumatic neuroses” OR "posttraumatic neurose" OR “posttraumatic neuroses” OR “traumatic distress” OR "posttraumatic psychose" OR “posttraumatic psychoses” OR "post traumatic psychose" OR “post traumatic psychoses” OR "posttraumatic syndrome" OR “posttraumatic syndrome” OR "post-traumatic syndrome" OR “post-traumatic syndromes” OR "trauma and stressor related disorder" OR “trauma and stressor related disorders” OR “traumatic stress disorder” OR “traumatic stress disorders” OR ptsd* OR ptss* OR “combat disorder” OR “combat disorders” OR “combat fatigue” OR “combat stress” OR “shell shock” OR “combat neurose” OR “combat neuroses” OR “war neurose” OR “war neuroses” OR “battle fatigue” OR “psychological stress” OR “psychological trauma” OR “psychological traumas” OR psychotrauma* OR “psycho-trauma” OR “trauma-focused” OR “psychological distress” OR “emotional trauma” OR “emotional traumas” OR “emotional distress” OR “emotional damage” OR “emotional injury” OR “mental damage” OR “mental harm” OR “mental injury” OR “mental trauma” OR “mental traumas” OR “sexual trauma” OR “psychological damage” OR “psychological harm” OR “psychological injury” OR “Posttraumatische Belastungsstörung” OR PTBS):ti,ab,kw | 14911 |
| #3 |  |  |
| #4 | #1 AND #2 AND #3 | 57 |
| #5 | #4 AND Trials | 55 |
