# Supplementary material for: Biological treatments for co-occurring eating disorders and psychological trauma: a systematic review
Source: Front Psychiatry. 2025 Feb 21;16:1523269. doi: 10.3389/fpsyt.2025.1523269 (PMC11914888; doi:10.3389/fpsyt.2025.1523269)
Supplement: Supplementary file 2 [file DataSheet2.docx]

|  | **Pre-intervention and at-intervention domains** | | | | | **Post-intervention domains** | | | | | | | | **Interpretation of domain-level and overall risk of bias** | | | | |
| --- | --- | --- | --- | --- | --- | --- | --- | --- | --- | --- | --- | --- | --- | --- | --- | --- | --- | --- |
| **Author and year** | **Bias due to confounding** | **Bias in selection of participants into study** | **Bias in classification of interventions** | | **Bias due to deviations from intended intervention** | | **Bias due to missing data** | | **Bias in measurement of outcomes** | | **Bias in selection of the reported result** | | **Within each domain** | | **Across domains** | **Criterion** | |  |
| Mahr et al., (2023) | Moderate | Moderate | Low | | Low | | Serious | | Serious | | Serious | | Moderate | | Serious | Serious | |  |
| Winkeler et al., (2022) | Low | Low | Low | | Low | | Low | | Low | | Low | | Low | | Low | Low | |  |
| Brewerton et al., (2022) | Moderate | Moderate | Low | | Low | | Serious | | Low | | Serious | | Moderate | | Serious | Serious | |  |
| Ragnhildstveit et al., (2021) | Serious | Serious | Low | | Low | | Low | | Low | | Serious | | Serious | | Serious | Serious | |  |
| Schwartz et al., (2021) | Serious | Serious | Low | | Low | | Moderate | | Moderate | | Moderate | | Serious | | Serious | Serious | |  |
| Woodside et al., (2021\|) | Low | Low | Low | | Low | | Moderate | | Moderate | | Moderate | | Low | | Moderate | Moderate | |  |
| Pacilio et al., (2019) | Serious | Serious | Low | | Moderate | | Serious | | Serious | | Serious | | Serious | | Serious | Serious | |  |
| Lipsman et al., (2017) | Low | Low | Low | | Low | | Serious | | Moderate | | Serious | | Low | | Serious | Serious | |  |
| Woodside et al., (2017) | Low | Low | Low | | Low | | Moderate | | Moderate | | Serious | | Serious | | Serious | Serious | |  |
| Tucker et al., (2004) | Moderate | Serious | Low | | Serious | | Serious | | Moderate | | Serious | | Serious | | Serious | Serious | |  |
| McCarthy et al., (1993) | Low | Serious | | Low | | Low | | Low | | Serious | | Serious | | Serious | Serious | | Serious | |
